# Detecting genotyping errors at *Schistosoma japonicum* microsatellites with pedigree information

**DOI:** 10.1186/s13071-015-1074-0

**Published:** 2015-09-08

**Authors:** Yu-Meng Gao, Da-Bing Lu, Huan Ding, Poppy H. L. Lamberton

**Affiliations:** Department of Epidemiology and Statistics, School of Public Health, Soochow University, Suzhou, 215123 China; Jiangsu Key Laboratory of Preventive and Translational Medicine for Geriatric Diseases, School of Public Health, Soochow University, Suzhou, 215123 PR China; Department of Infectious Disease Epidemiology, Imperial College London, London, W2 1PG UK

**Keywords:** *Schistosoma japonicum*, Microsatellite, Genotyping errors, Pedigree

## Abstract

**Background:**

Schistosomiasis japonica remains a major public health problem in China. Integrating molecular analyses, such as population genetic analyses, of the parasite into the on-going surveillance programs is helpful in exploring the factors causing the persistence and/or spread of *Schistosoma japonicum*. However, genotyping errors can seriously affect the results of such studies, unless accounted for in the analyses.

**Methods:**

We assessed the genotyping errors (missing alleles or false alleles) of seven *S. japonicum* microsatellites, using a pedigree data approach for schistosome miracidia, which were stored on Whatman FTA cards.

**Results:**

Among 107 schistosome miracidia successfully genotyped, resulting in a total of 715 loci calls, a total of 31 genotyping errors were observed with 25.2 % of the miracidia having at least one error. The error rate per locus differed among loci, which ranged from 0 to 9.8 %, with the mean error rate 4.3 % over loci. With the parentage analysis software Cervus, the assignment power with these seven markers was estimated to be 89.5 % for one parent and 99.9 % for a parent pair. One locus was inferred to have a high number of null alleles and a second with a high mistyping rate.

**Conclusion:**

To the authors’ knowledge, this is the first time that *S. japonicum* pedigrees have been used in an assessment of genotyping errors of microsatellite markers. The observed locus-specific error rate will benefit downstream epidemiological or ecological analyses of *S. japonicum* with the markers.

**Electronic supplementary material:**

The online version of this article (doi:10.1186/s13071-015-1074-0) contains supplementary material, which is available to authorized users.

## Background

Whilst there have been great successes in the control of schistosomiasis japonica in China over the last six decades, the disease remains a major public health problem with an estimated 0.29 million people infected and over 245 million people living in endemic areas [1]. Moreover, the disease has been resurging in areas where it was previously well controlled or its transmission interrupted [2, 3]. Therefore, it is of importance to explore the factors influencing the persistence and/or spread of *Schistosoma japonicum*. Molecular approaches, for example population genetic analyses, can be applied and integrated into the on-going parasitological or serological surveillance programs [4–6] to enhance our knowledge of transmission of this disease, including addressing questions such as who is infecting whom?

Population genetic analyses of parasites can elucidate parasite transmission patterns by understanding gene flow and population structure between and among spatial or temporal parasite populations [7–9]. However, as adult worms reside in the blood veins of mammalian hosts and sampling the worms directly from live hosts is impossible, studying population structure of schistosomes in the field is logistically challenging. Therefore the alternative practice is to collect and genotype schistosome larvae: either miracidia hatched from eggs in host faeces, or cercariae shed from an intermediate host snail. This is facilitated by the development of the Whatman FTA card-based approach [10], which allows field-collected larvae to be stored at room temperature for up to 4 years and to be successfully reused for molecular analyses up to 11 times [11]. This in turn facilitates the growing research in the molecular epidemiology of *S. japonicum* [12–14, 11].

Genotyping errors (i.e. the proportion of observed alleles or genotypes which differ from the true alleles or genotypes) can bias the frequencies reported for a population. Even a small per-locus genotyping error rate can result in relatively large probabilities of a multilocus genotype containing at least one error [15, 16]. Hence, there is an increasing call for reporting genotyping error rates and then integrating these errors in the downstream population genetic analyses [17, 18, 15]. Several approaches have been proposed for the quantification of genotyping errors [19], among which, detecting errors with pedigree data has been the most robust assay [17]. Therefore, we assessed the genotyping errors on seven commonly used *S. japonicum* microsatellites [20–22], as seen in Table 1, for schistosome miracidia stored on Whatman FTA cards. Our *S. japonicum* pedigree was established from laboratory crosses of parasites, with adult worm pairs, and their offspring (miracidia) from each parasite family, collected. Each parasite was genotyped in an individual multiplex PCR reaction. Genotyping errors in offspring with the seven microsatellite loci were detected based on Mendelian inheritance of alleles, and further estimated with the widely used program CERVUS V3.0.7 [23]. The results benefit future molecular analyses of this organism, aiding accurate interpretation of results from amplification using these common markers.

**Table 1:** Characteristics of seven microsatellite loci on S. japonicum reported in the literature

Locus	Primer sequence (5’-3’)	Repeat	Schistosome isolates	Number of alleles	Size range (bp)	He	Reference
Sjp4	F: ACAAGCTCCAATCGTCTCTGA	TAA	Five provinces^a^, China	20	190-247	0.62–1.00	Yin, et al. [22]
	R: GAATACTGCCGCCCTTGTAA						
Sjp18	F: TCCTTTATCTGGGCTGTGGA	TGA	Two provinces^b^, China	7	261–298	0.68	Xiao, et al. [21]
	R: TTTCAGCAGGATAACATGACG						
Sjp22	F: CAAAGCCTAAACGTCATAGACAG	TTA	Two provinces^b^, China	11	105–167	0.85	Xiao, et al. [21]
	R: CAACCACCGATAAGTAGAGTGGA						
Sjp42	F: GCTGCAGCTTCTGTGTAGTAA	TAA	Two provinces^b^, China	9	199–234	0.95	Xiao, et al. [21]
	R: GTCTTGCTCAGATCAGTTCGT						
Sjp58	F: TCCCAGTACCAATGTAGATGTG	AAT	Two provinces^b^, China	12	439–499	0.8	Xiao, et al. [21]
	R: CTAATAAAGTCGTCAAGGAGCA						
Sjp60	F: CGATTCATTCATAGCCTGACT	TAT	Two provinces^b^, China	10	134–165	0.9	Xiao, et al. [21]
	R: GAATCCCATCACAGATTAACG						
TS2	F: TTGTCAATAATTTCACTAGGTTCAC	GT	The Philippines and two provinces^c^, China	5	360–385	0.68	Shrivastava, et al. [20]
	R: AATTAATAATTCACAAGTAAAACATCTAAGT						

He, unbiased expected heterozygosity. ^a^Five provinces are Anhui, Jiangxi, Hunan, Hubei and Sichuan. ^b^Two provinces are Anhui and Hubei.^c^Two provinces are Anhui and Zhejiang

## Methods

### Parasite pedigrees

*Schistosoma japonicum* was originally obtained from infected snails from the Shitai county of Anhui, China in April 2013 and then maintained in mice in the laboratory. Miracidia were hatched from eggs collected from the livers of mice. Individual snails, with no previous schistosome infections, were individually exposed to a single miracidium and later checked for infection of schistosome with a shedding experiment. For the details in procedure, see the work in [24, 25]. As the parasite undergoes only asexual reproduction within snail hosts these individually laboratory-infected snails therefore each harbored only clonal cercariae of the same gender and genotype. Twenty mice were individually exposed to approximately 50 cercariae from only one snail each (i.e. one mouse per snail) and the resultant adult worms were morphologically sexed, to back inform on the sex of the cercariae infection in each snail. The sex of cercariae from 11 single miracidium-exposed snails were successfully identified, with six snails harboring female cercariae and five snails harboring male cercariae.

**Fig. 1:**
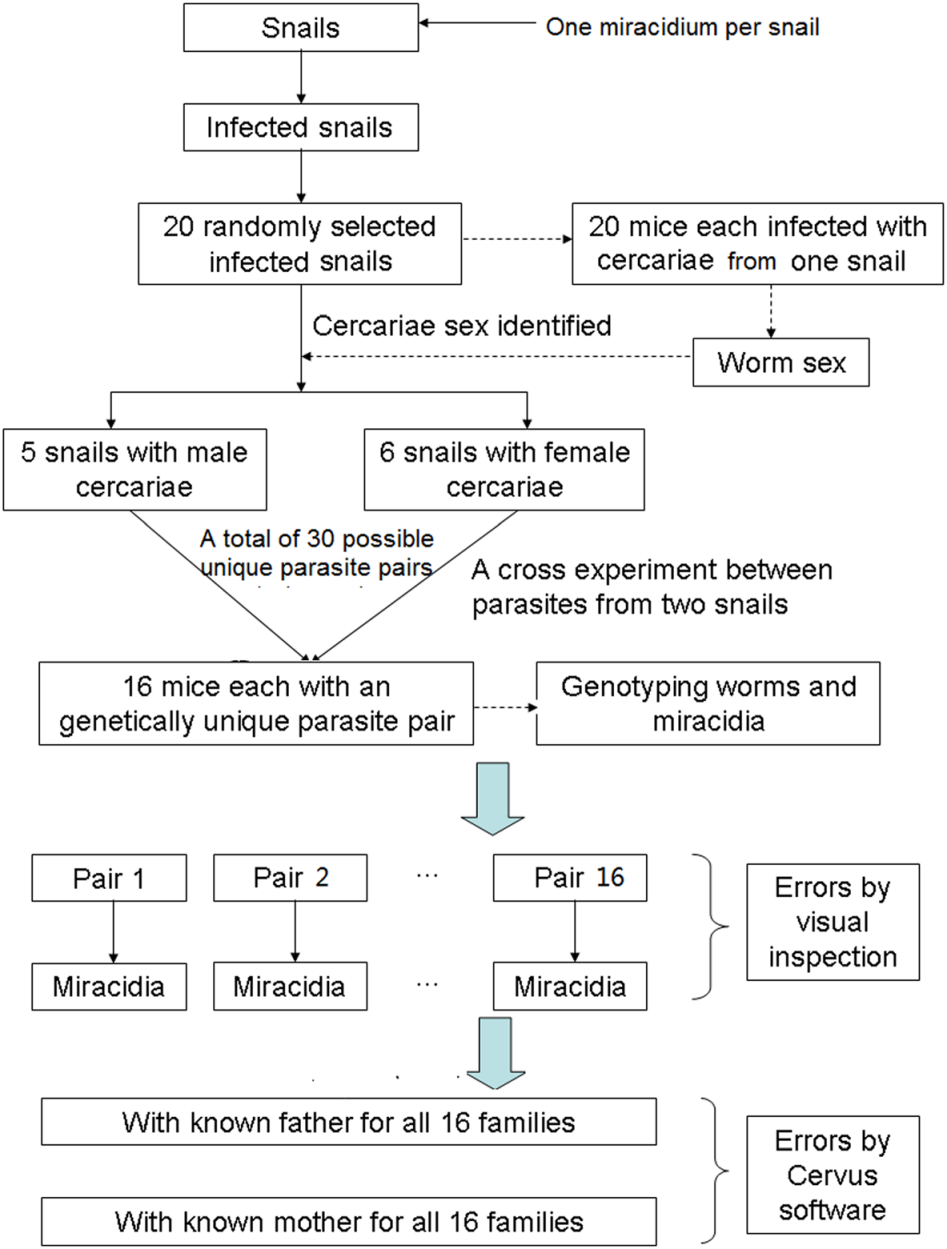
Diagrammatic representation of experimental steps and molecular analyses

The cercariae from these 11 snails were used for parasite cross experiments. Sixteen mice were each exposed to two genotypes of five cercariae from two snails, i.e. five male cercariae of the same genotype from one snail and five female cercariae of the same genotype from another snail (one mouse per unique genotype cross). Due to limited cercariae shed from the 11 infected snails and to minimize animal usage, only a total of 16 genetically unique worm pairs were established in 16 mice, rather than the theoretically possible 30 unique worm pairs from 5 male and 6 female parasite clones (Fig. 1). Adult worm pairs were obtained six weeks post-exposure via portal perfusion and liver examination of the mouse. The adult worm pairs were stored in 99 % ethanol and frozen for future analyses. The liver was minced and eggs (i.e. the offspring of the genetically unique adult worm pairs) were collected for hatching of miracidia. The larvae, with the small size of 150 μm [26], were then collected individually using a pipette in 3–5 μl water and stored on a Whatman FTA Classic Card for subsequent DNA analysis. A total of 16 different parasite families with known adult worm pairs and their offspring were obtained for molecular analyses. The experiments including the following molecular analyses are shown in Fig. 1. Ethical Approval: The research was approved by the Soochow University Ethics Committee and the care and use of experimental animals complied with institutional standards.

### DNA extraction and microsatellite amplification

Genomic DNA from adult worms was extracted using an EZgene™ Mollusc gDNA Kit (Biomiga, Inc. San Diego, USA) according to the manufacturer’s protocols. DNA extraction from miracidia was performed as described elsewhere [10]. A total of seven previously published microsatellite loci were investigated, and the forward primer for each pair was labeled with 6-FAM, HEX, TAMRA or ROX (Table 2). PCR reactions were carried out in 15 μl reaction volumes containing 1 μl of adult worm DNA or from one FTA Whatman card disc with a single miracidium, using the QIAGEN Multiplex PCR Kit (cat. nos. 206152, Germany). Thermo cycling was carried out in an Arktik thermocycler (Thermo Scientific) with the following PCR profile: 95 °C for 5 min, followed by 40 cycles of 30s at 95 °C, 90s at annealing temperature (five cycles at each temperature from 57 °C to 51 °C decreased by 2 °C, then 20 cycles at 50 °C), and 30s at 72 °C, with a final extension at 68 °C for 10 min. Multiplexed PCR products were genotyped using an ABI 3100 automated sequencer (Applied Biosystems) in Sangon Biotech (Shanghai, China). Each adult worm was multiplexed twice to improve accuracy of true genotype scoring.

**Table 2:** Characteristics of *S. japonicum* adult worms (five males and six females) and offspring miracidia genotyped at seven mircrosatellite loci in this study

		Adult worms			Offspring miracidia		
Locus	Dye	No. amplification	No. alleles	Ho	No. amplification	No. alleles	Ho
Sjp4	Hex	11	3	0.545	107	3	0.523
Sjp18	Tamra	11	7	0.727	107	7	0.841
Sjp22	Hex	11	4	0.273	102	5	0.402
Sjp42	Fam	9	6	0.667	103	6	0.515
Sjp58	Tamra	11	6	0.636	96	6	0.625
Sjp60	Tamra	11	8	0.727	105	8	0.867
TS2	Rox	11	4	0.455	95	5	0.474

*Ho* observed heterozygosity

### Microsatellite scoring

We combined automated allele calling with visual inspection of each sample. GeneMarker HID Version 2.6.1 (SoftGenetics LLC) was used to automatically score alleles. The parameters and the loci bin ranges were set based on *S. japonicum* samples from several geographical regions to maximize the match to the characteristics of the loci used. Automated binning of allele data (i.e. converting the raw decimal data into integers) provided consistency across multiple plates of PCR products, whereas visual inspection avoided the errors due to uncorrected size measure with low scores shown in the software. See Additional file 1 for an example.

### Identification and quantification of genotyping errors

There are three common types of genotyping errors including: 1) null allele, a non-amplifying allele due to a mutation in the primer target sequence [27]; 2) allelic dropout, the stochastic non-amplification of an allele at a heterozygous locus [28]; 3) false allele, allele-like PCR-generated artifact [29]. For practical handling and as in the work [30], two types of errors, missing alleles (i.e. generally caused by the existence of an allelic dropout or a null allele) and false alleles, were classified here and identified through Mendelian-inheritance checking. A missing allele is an allele that is not observed in a miracidium but is expected to be inherited from a parent adult worm. A false allele is an allele that is called from a miracidium, but does not exist in either parent worm. This could result from a mutation between two generations or a false peak [31]. If there was no amplification at all on a given loci, then we did not include this as a ‘non amplification’ event as in this situation, no allele calls would be made at all, as the amplification has not worked for that loci, and therefore this scenario would not bias downstream molecular analyses. As recommended in [32], we calculated two indexes, error rate per locus and per multilocus genotype. Error rate per locus is measured as the ratio between the number of single-locus genotypes including at least one allelic mismatch and the number of single-locus genotypes examined, calculated for each locus and over loci. Error rate per multilocus genotype is the ratio between the number of multilocus genotypes including at least one allelic mismatch and the number of multilocus genotypes examined (i.e. the miracidia error rate).

Cervus V3.0.7 [23, 33], a program for parentage analysis, was used to calculate indexes including Number of alleles, Observed heterozygosity (Ho), Expected heterozygosity (He), Polymorphic information content (PIC), Average non-exclusion probability of the first parent (Excl1), Average non-exclusion probability of parent pair (Excl2), and Null allele frequency, for each locus and over all loci (if allowable). Numbers of mismatches were also detected in known mother-offspring or known father-offspring pairs for each locus. The mistyping rate was calculated as the ratio of the number of mismatches to the number of alleles compared, scaled by the average probability of detecting a mismatch [23].

## Results

### Parasite profile

As seen in Table 2, an average of 6.7 miracidia from each genetically unique adult worm pair (i.e. a total of 107 offspring miracidia from 16 mice) were successfully genotyped. The number of alleles ranged from 3 to 8 among loci with an average of 5.43 per locus in the 11 parental schistosomes (five male and six female) and of 5.71 per locus in the 107 miracidia. The observed heterozygosity was between 0.273 and 0.727 in the parental worms and between 0.402 and 0.867 in the miracidia. The value of no amplification rate in miracidia varied among loci, ranging from 0 for Sjp4 and Sjp18 to 11.2 % (12/107) for TS2. All genotype data were seen in Additional file 2.

### Genotyping errors

From Table 3, a total of 31 genotyping errors, with 29 missing alleles and two false alleles, were detected in 107 miracidia individuals through visual inspection of incompatibility between worm pairs and their offspring. The error rate per locus differed among loci, with the highest of 9.8 % for Sjp22. The mean error rate over loci was 4.3 %. Among 107 miracidia multilocus genotypes checked, 27 individuals had at least one genotyping error, which gave an error rate of 25.2 % per multilocus genotype.

**Table 3:** Locus-specific genotyping error rates in 107 miracidia individuals identified from incompatibility between parent pairs and their offspring

Locus	No. genotype examined	No. missing alleles	No. false alleles	Total errors	Error rate
Sjp4	107	0	0	0	0
Sjp18	107	0	0	0	0
Sjp22	102	10	0	10	0.098
Sjp42	103	3	0	3	0.029
Sjp58	96	4	1	5	0.052
Sjp60	105	6	0	6	0.057
TS2	95	6	1	7	0.074
Overall		29	2	31	0.043

### Parentage analyses with the software Cervus

As required by Cervus, we combined both 11 worms and 107 miracidia to estimate the genetic diversity and probabilities of parent exclusion in parentage analyses. As seen in Table 4, the unbiased expected heterozygosity (He) ranged from 0.493 to 0.830 among loci, with the mean 0.652 over loci. The mean PIC was up to 0.603 over loci. When the seven loci were combined, the average non-exclusion probability of the first parent (Excl1) and the average non-exclusion probability of the parent pair were 89.5 % and 99.9 %, respectively. Null allele frequency was less than 0.05 for all loci with the exception of TS2.

**Table 4:** Genetic diversity estimates, probabilities of parental exclusion and frequency of null alleles for the seven loci on adult worms and offspring analyzed with CERVUS3.07

Locus	No. alleles	Ho	He	PIC	Excl1	Excl2	Null freq
sjp4	3	0.525	0.493	0.399	0.880	0.670	−0.037
sjp18	7	0.831	0.732	0.692	0.668	0.295	−0.069
sjp22	5	0.389	0.575	0.517	0.828	0.513	0.185
sjp42	6	0.527	0.702	0.664	0.700	0.320	0.135
sjp58	6	0.626	0.666	0.636	0.728	0.332	0.033
sjp60	8	0.853	0.830	0.807	0.508	0.157	−0.016
TS2	5	0.472	0.568	0.509	0.831	0.516	0.090
Mean/Total			0.652	0.603	0.895	0.999	

*Ho* observed heterozygosity, *He* unbiased expected heterozygosity, *PIC* polymorphic information content, *Excl1* one parent exclusion, *Excl2* two parent exclusion

As seen in Table 5, a total of 16 mismatching calls were detected in known mother-offspring pairs and 15 in known father-offspring pairs. No mistyping was identified for either Sjp4 and Sjp18. The highest mistyping rate of up to 11.4 % in mother-offspring and 17.1 % in father-offspring was estimated for the locus Sjp22. The second highest was observed for the locus TS2.

**Table 5:** Locus-specific genotyping error rates estimated from mismatches between adult females or males and offspring with the program CERVUS3.07

Locus	Detection probability	Mother-offspring pairs			Father-offspring pairs		
		No. genotypes compared	No. alleles mismatching	Estimated error rate	No. genotypes compared	No. alleles mismatching	Estimated error rate
sjp4	0.120	107	0	0	107	0	0
sjp18	0.332	107	0	0	107	0	0
sjp22	0.172	102	4	0.114	102	6	0.171
sjp42	0.300	103	3	0.049	76	0	0
sjp58	0.272	96	3	0.057	96	2	0.038
sjp60	0.492	105	3	0.029	105	3	0.029
TS2	0.169	95	3	0.093	95	4	0.124

Detection probability, the average probability of exclusion of a single randomly-chosen unrelated individual from parentage when no information is available from the known parent. Estimated error rate, the ratio of the number of mismatches to the number compared scaled by the average probability of detecting a mismatch

## Discussion

We evaluated the genotyping errors in individual multilocus reactions involving seven *S. japonicum* microsatellite markers for miracidia samples stored on Whatman FTA cards. Our results can inform future studies using these microsatellites through a more thorough understanding of the potential error rates of multiplex amplifications, aiding accurate interpretation of their results. Among the microsatellites used, two had 100 % success rate in amplification and no errors recorded, indicating these as highly reliable and reproducible markers and therefore highly recommended for future use. Two loci, one with the highest mistyping rate and one with possible null alleles were identified, and should be used with caution under these PCR conditions in the future. To the authors’ knowledge, this is the first time that *S. japonicum* pedigrees have been used in an assessment of genotyping errors with microsatellite markers.

The selected set of seven schistosome microsatellites revealed high genetic variation in the 11 parasites (five male and six female) used and their offspring miracidia. If there were no genotyping errors, their considerably high polymorphism and combined assignment power would be very useful in parentage analyses, and then can be used to track transmission of the parasite [34] and other population genetic analyses. In the current study, for practical handling and due to the approach of sample storage we classified the observed genotyping errors into two types- missing and false alleles. Using pedigree analyses, we observed up to one-fourth of miracidia individuals could have at least one error, which is much lower than the proportion (44 %) reported for *S. mansoni* [35]. One possible reason would be associated with more loci (i.e., nine) used in the *S. mansoni* study.

An error rate of 2 % in microsatellite studies is usual and acceptable [36, 15]; however, in this study we observed a considerably high mean error rate over loci (4.3 %). The error rate varied with locus. No errors were observed at the loci Sjp4 and Sjp18. Particularly for Sjp18, a high polymorphism was also displayed in the samples used, indicating that this microsatellite marker is highly informative for this kind of analyses. However, at the locus Sjp22, one-third of the total errors were detected, plus one false allele observed, therefore, special precautions should be taken in downstream analyses of the molecular data created from this locus.

Cervus is a likelihood-based parentage-assignment program [23, 33] and has been the most widely employed in inferring parent–offspring relationships [37]. With known pedigree data (for example, a known parent-offspring pairing) the software can also be used to quantify mistyping errors and then estimate error rate. In this study with Cervus, two loci (Sjp22 and TS2) were shown to contain high errors and the locus TS2 was suggested with potential null alleles. To minimize genotyping errors, a protocol implementing quality assurance procedures has been proposed [17], but genotyping errors cannot be completely eliminated. Therefore the knowledge of errors, particularly the locus-specific error rate, can add power and accuracy to downstream analyses, especially as a majority of software packages, for example Colony2 [38] and MasterBayes [39], have been developed to allow the incorporation of errors into their algorithms. Our data are also important for future optimization of improved multiplex reactions, indicating certain loci which are highly reliable (Sjp4 and Sjp18), and other microsatellites which may be either improved or replaced (Sjp22 or TS2).

Overall false alleles were rare, indicating that if allele calls are made for these loci, then they are likely to be accurate. However some alleles may be lost. Overall this means that any bias that genotyping error may impose will be more likely to be associated with genetic diversity, rather than population structure analyses. This may increase the relative proportion of homozygous calls versus heterozygous calls, as well as reducing the number of rare and private alleles. However, if the same multiplex reactions are used to compare populations, then a similar loss of diversity would be expected across the samples and should not affect the overall conclusions. As this study was performed comparing miracidia directly with each other and their parents, the chance of these differences we observe and attribute to amplification and genotyping errors, being due to mutations arising from only one round of sexual reproduction, is minimal, particularly given the relatively low mutation rate of schistosomes [40]. We are therefore confident that our results represent accurate measures of genotyping errors for each loci.

## Conclusion

The genotyping errors of *S. japonicum* at seven loci were characterized with pedigree data. Two error-prone loci were identified and should be paid more attention. Null alleles at one locus were detected with the program Cervus. The observed locus-specific error rate is useful for any further epidemiological, ecological or evolutionary research on *S. japonicum* involving the above microsatellite markers.

## Additional files

Additional file 1:
**An example for allele calling with GeneMaker HID V2.6.1 Demo.** (DOC 3162 kb) Additional file 2:
**Genotype data on offspring miracidia and their parental worms.** (XLS 48 kb)
