# Supplementary material for: Detecting genotyping errors at Schistosoma japonicum microsatellites with pedigree information
Source: Parasit Vectors. 2015 Sep 8;8:452. doi: 10.1186/s13071-015-1074-0 (PMC4563838; doi:10.1186/s13071-015-1074-0)

**Additional file 1. An example of allele calling with GeneMarker HID V2.6.1 Demo**

1.Run GeneMarker HID V2.6.1 Demo

We crated specific panels for *S. japonicum*, and all original panels for humans have been deleted.


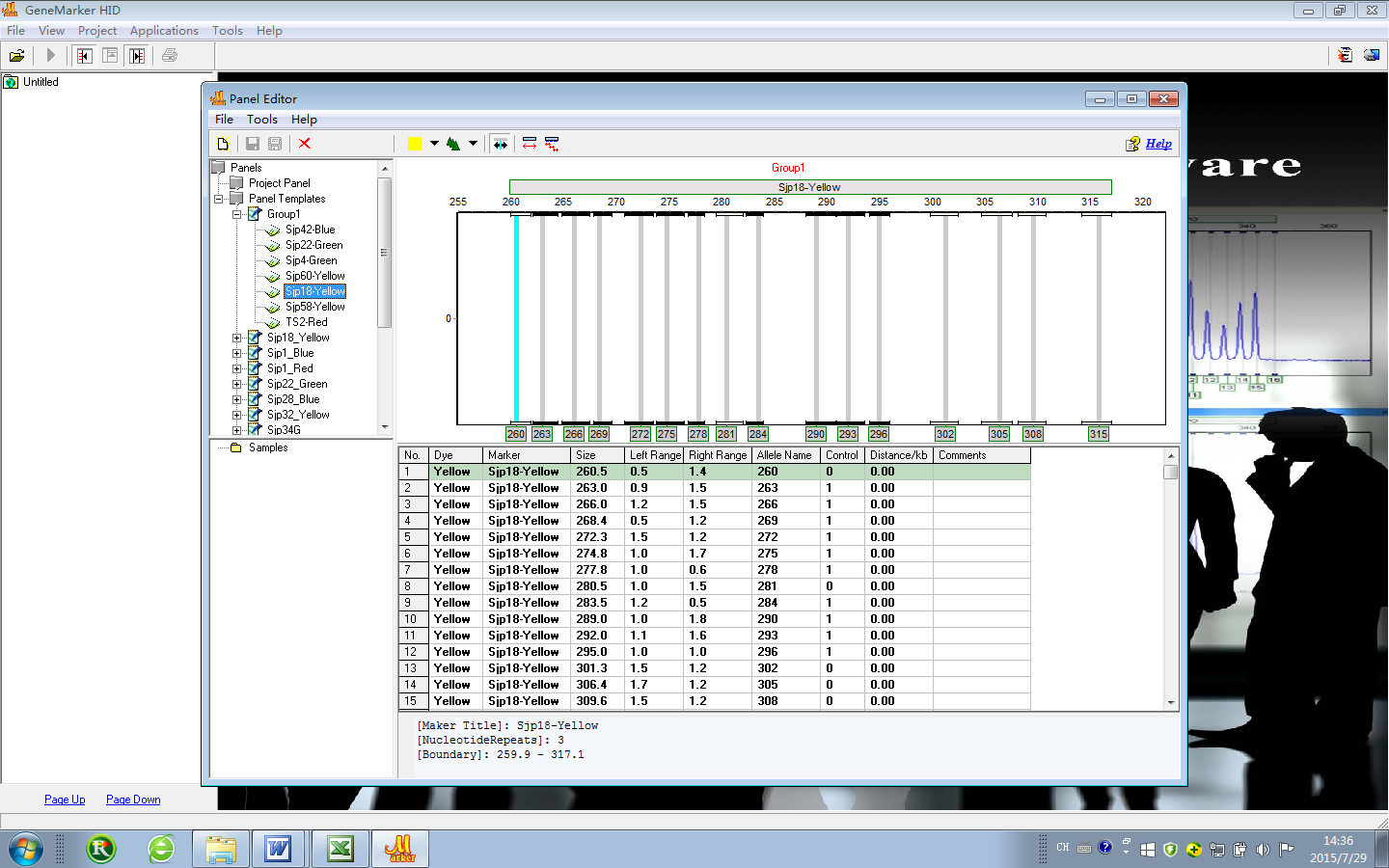


2.Example Data. Please also see Additional file 2.

Parents:

File 2014-12-11_G09_**G09**, for B4 (female worm, mother)

File 2014-12-11_H03_**H03**, for C2 (male worm, father)

Offspring miracidia:

File 2014-10-10_A09_**A09**, for miracidia B4C2-1

File 2014-10-10_A10_**A10,** for miracidia B4C2-2

File 2014-10-10_A12_**A12**, for miracidia B4C2-4

File 2014-10-10_B01_**B01**, for miracidia B4C2-5

File 2014-10-10_B02_**B02**, for miracidia B4C2-6

File 2014-10-10_B03_**B03**, for miracidia B4C2-7

File 2014-10-10_B04_**B04**, for miracidia B4C2-8

3. Input Example Data B4C2 and run Panel Sjp18

Checking scores for each sample, which all were over 90.


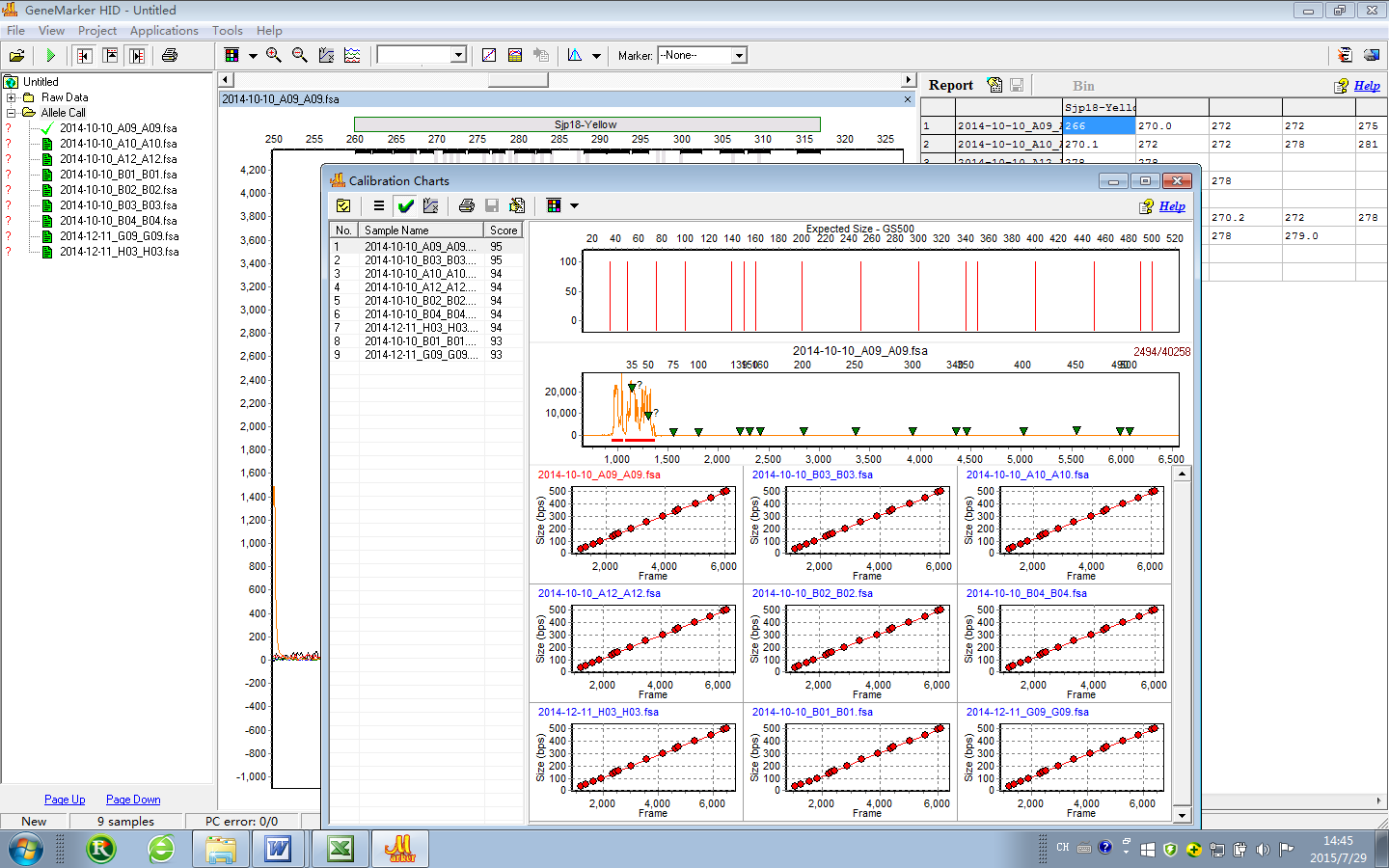


4. Allele calling at locus Sjp18

1) miracidia B4C2-1: 272, 278


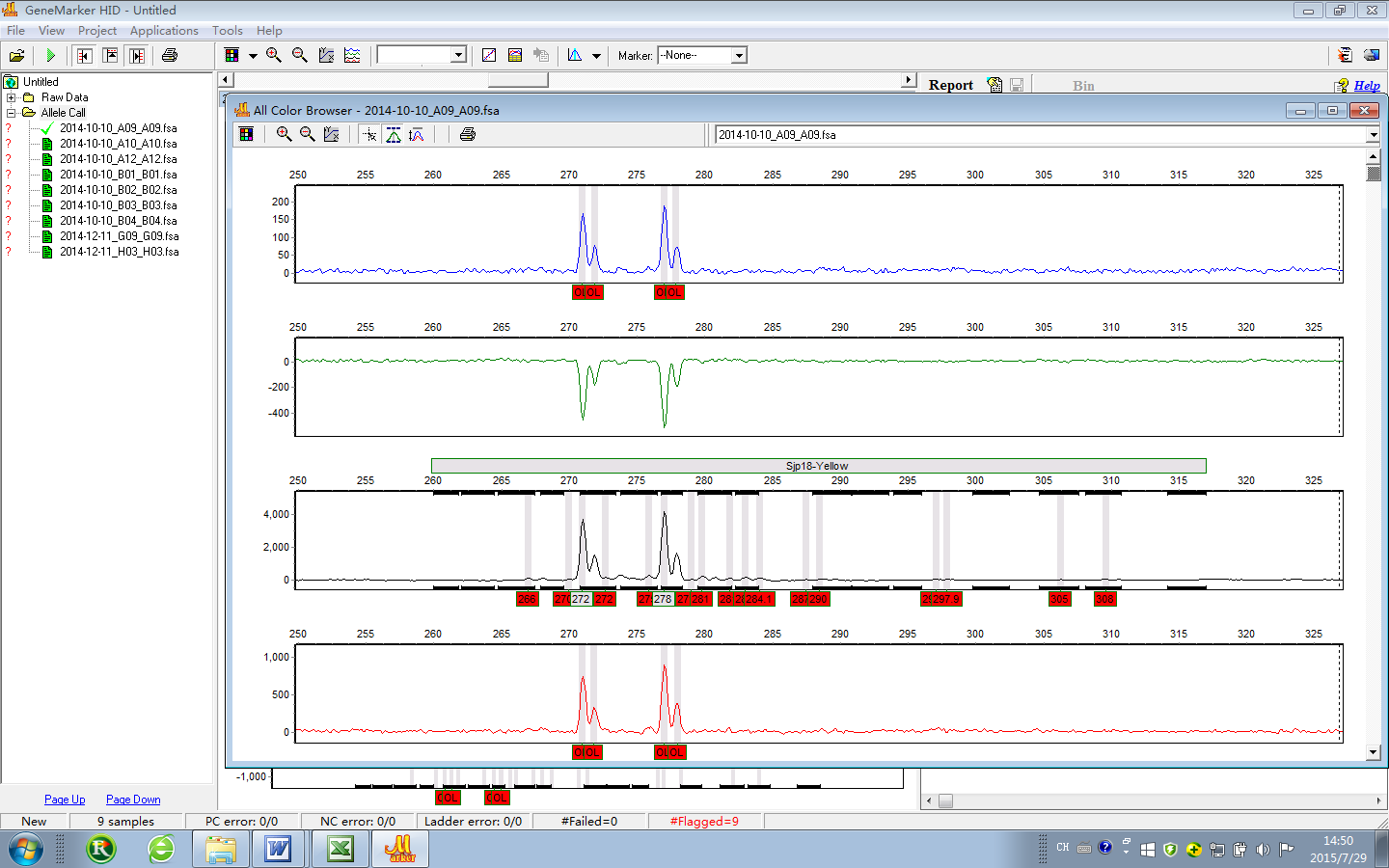


2) miracidia B4C2-2: 272, 278


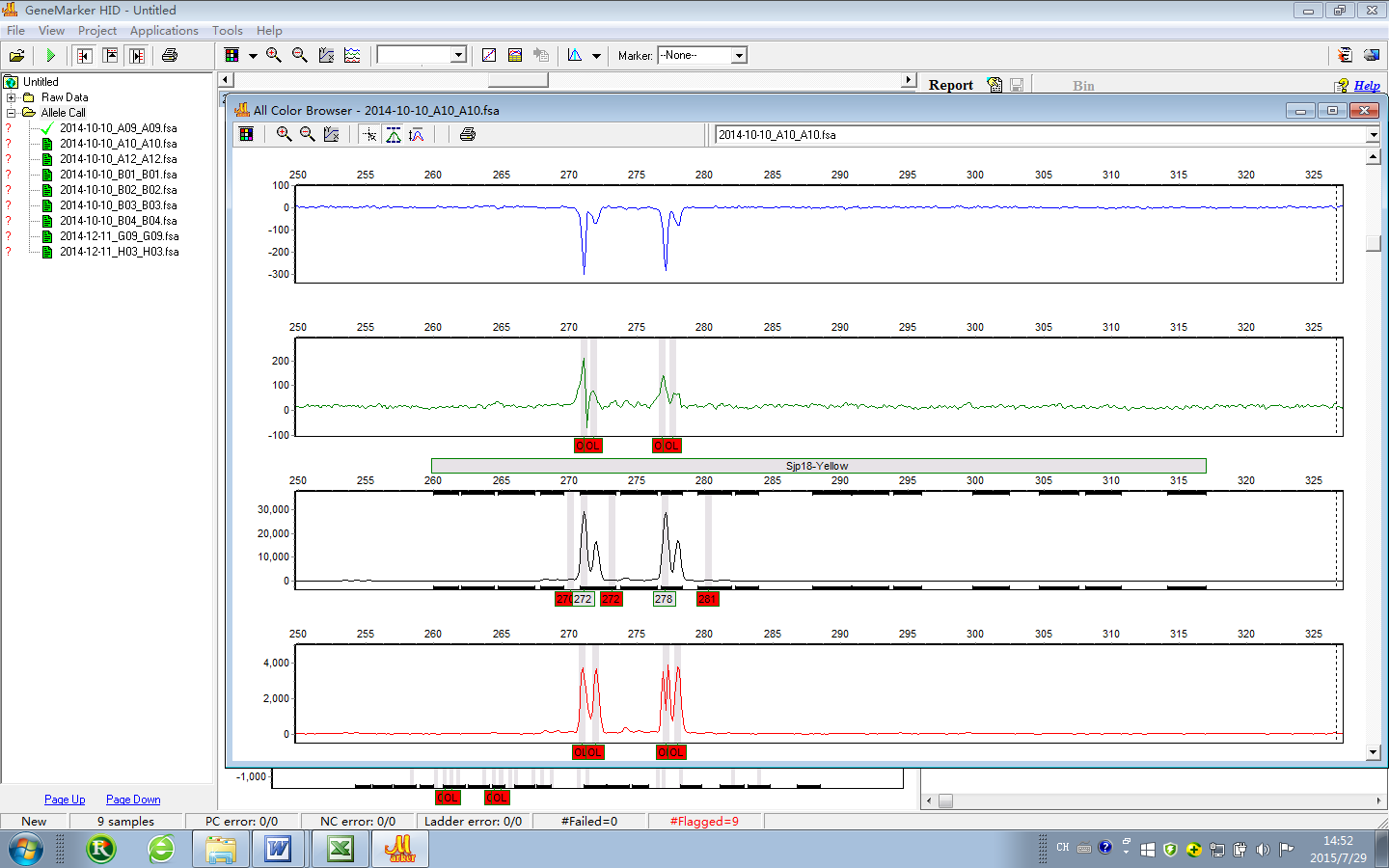


3) miracidia B4C2-4: 278


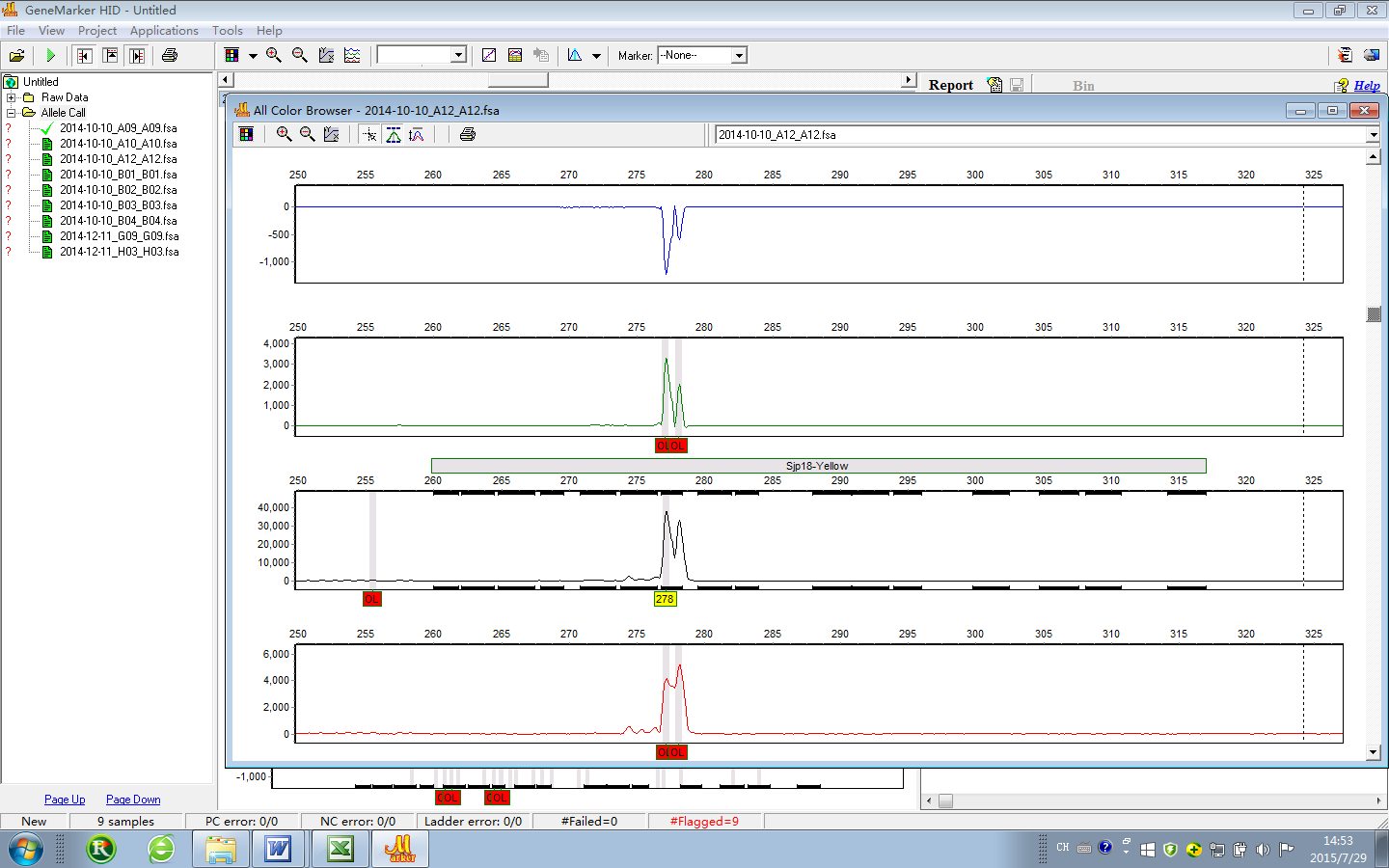


4) miracidia B4C2-5: 272, 278


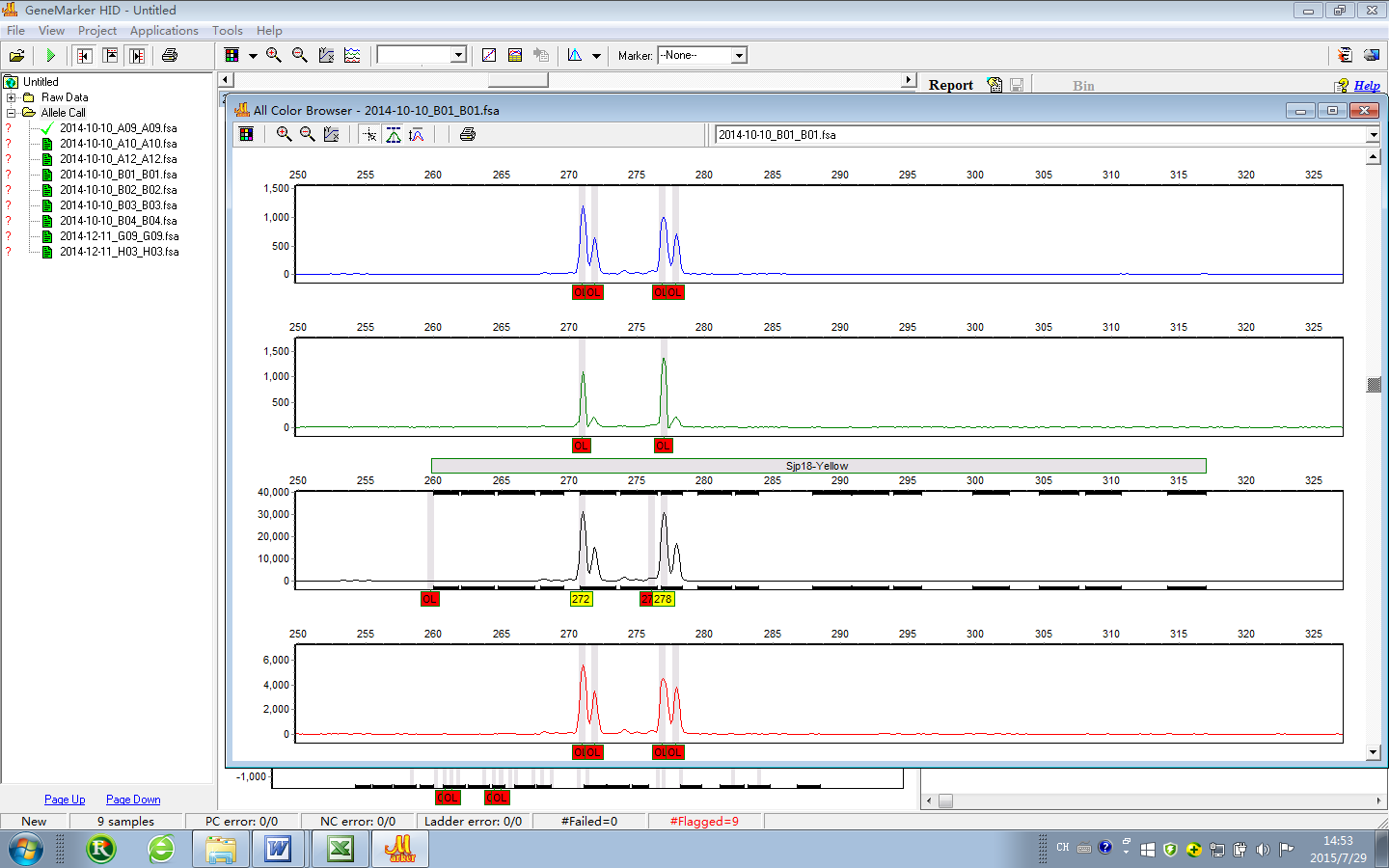


5) miracidia B4C2-6: 278


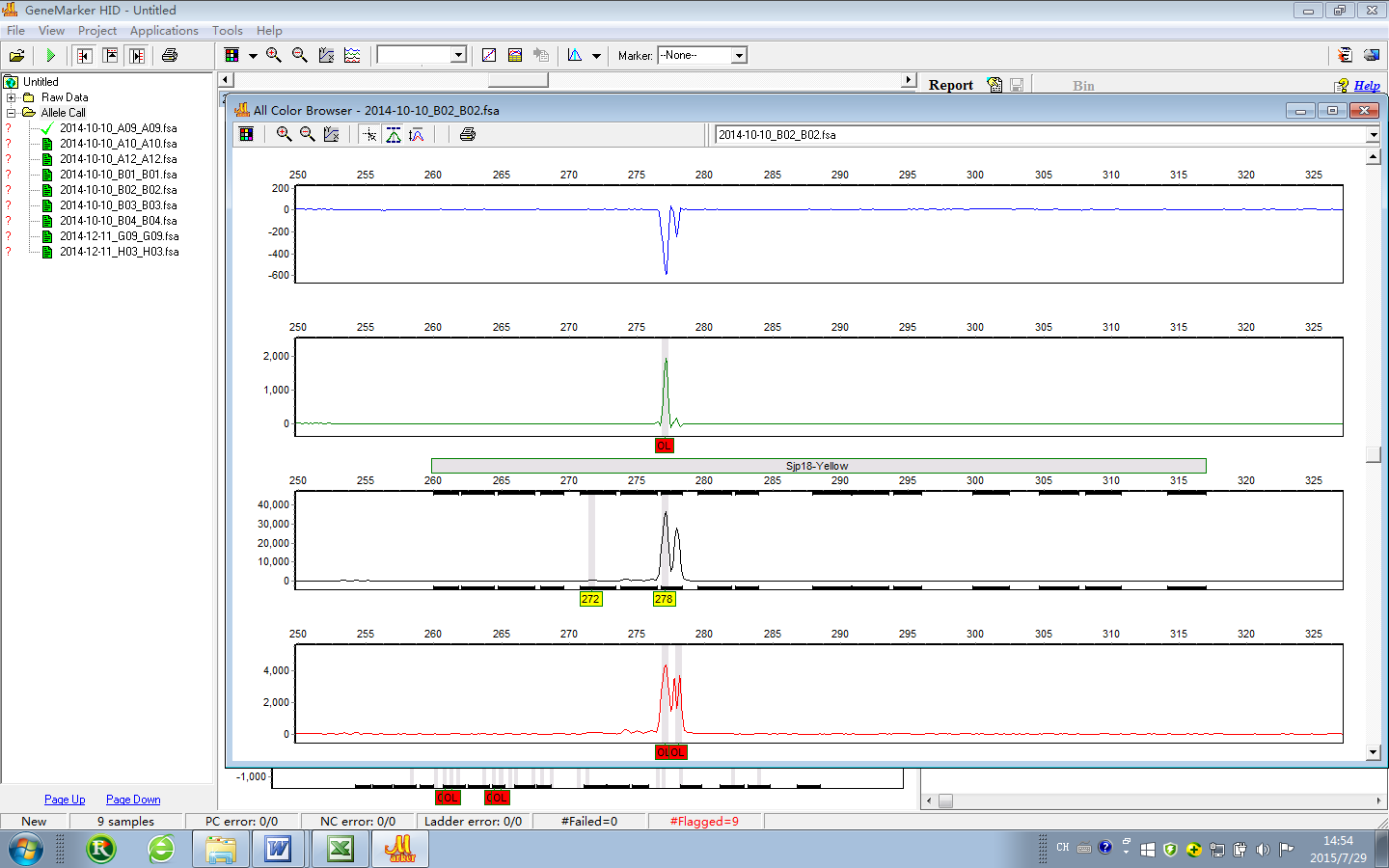
 6) miracidia B4C2-7: 272, 278


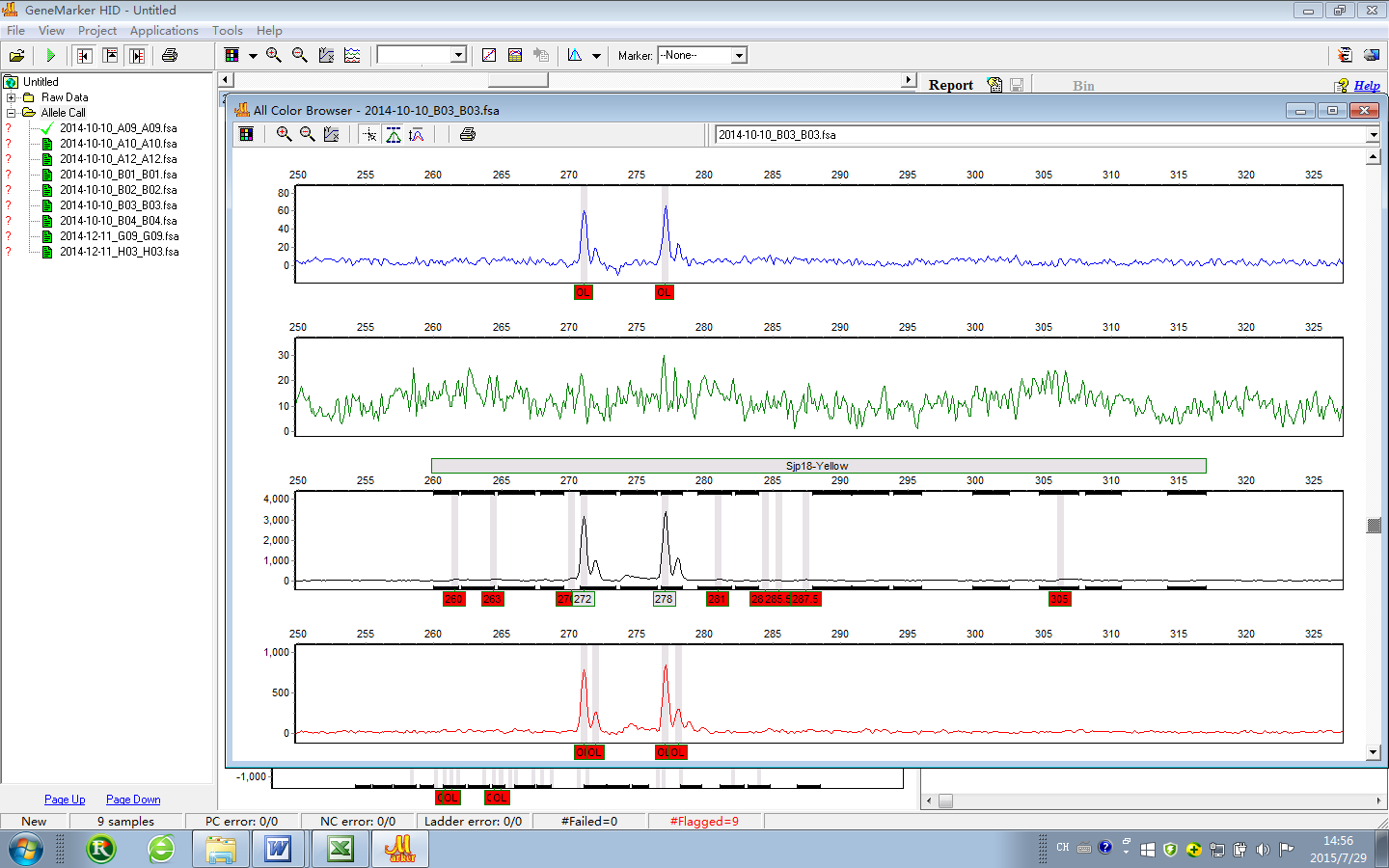


7) miracidia B4C2-8: 272, 278


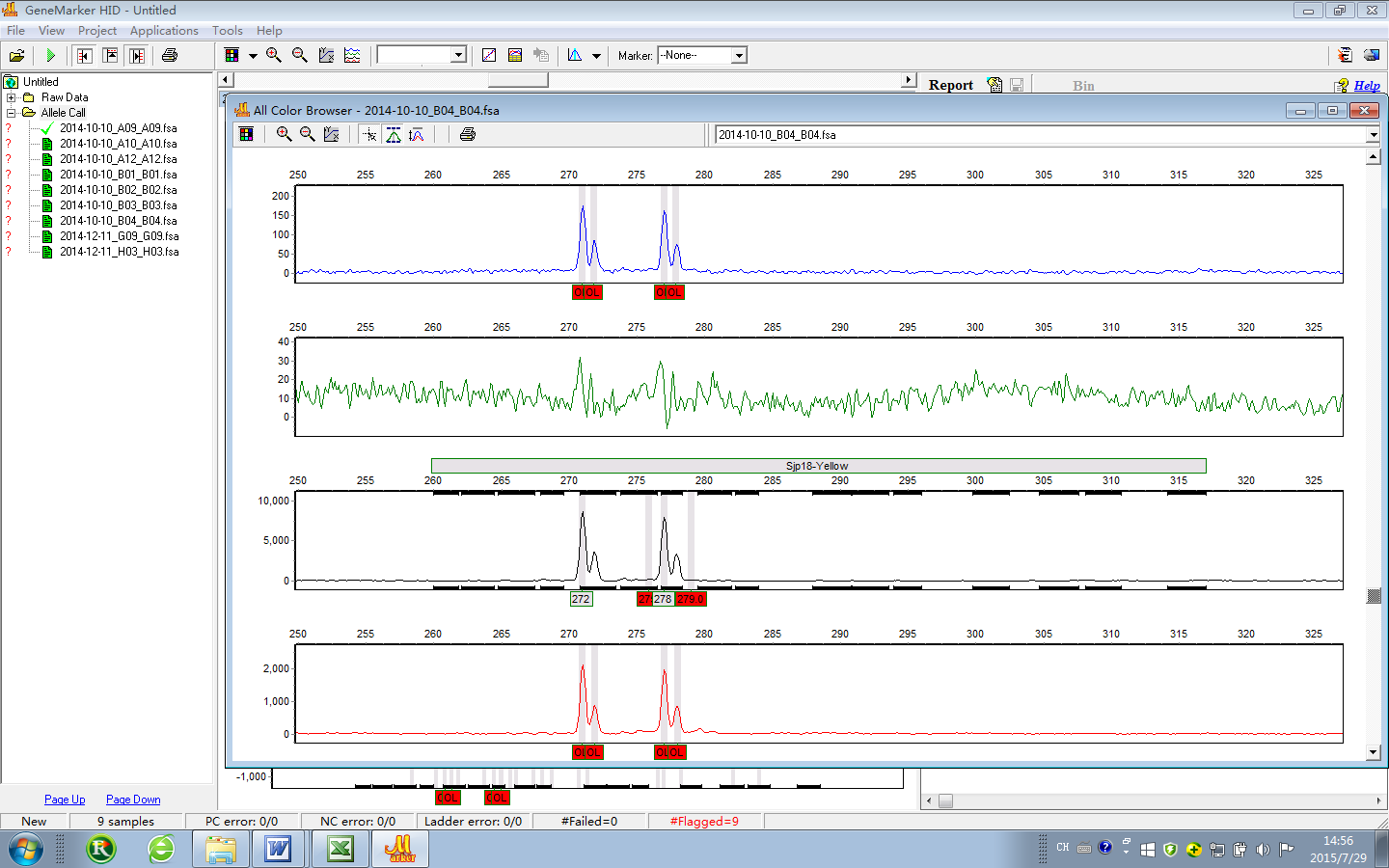


8) B4, male worm: 278


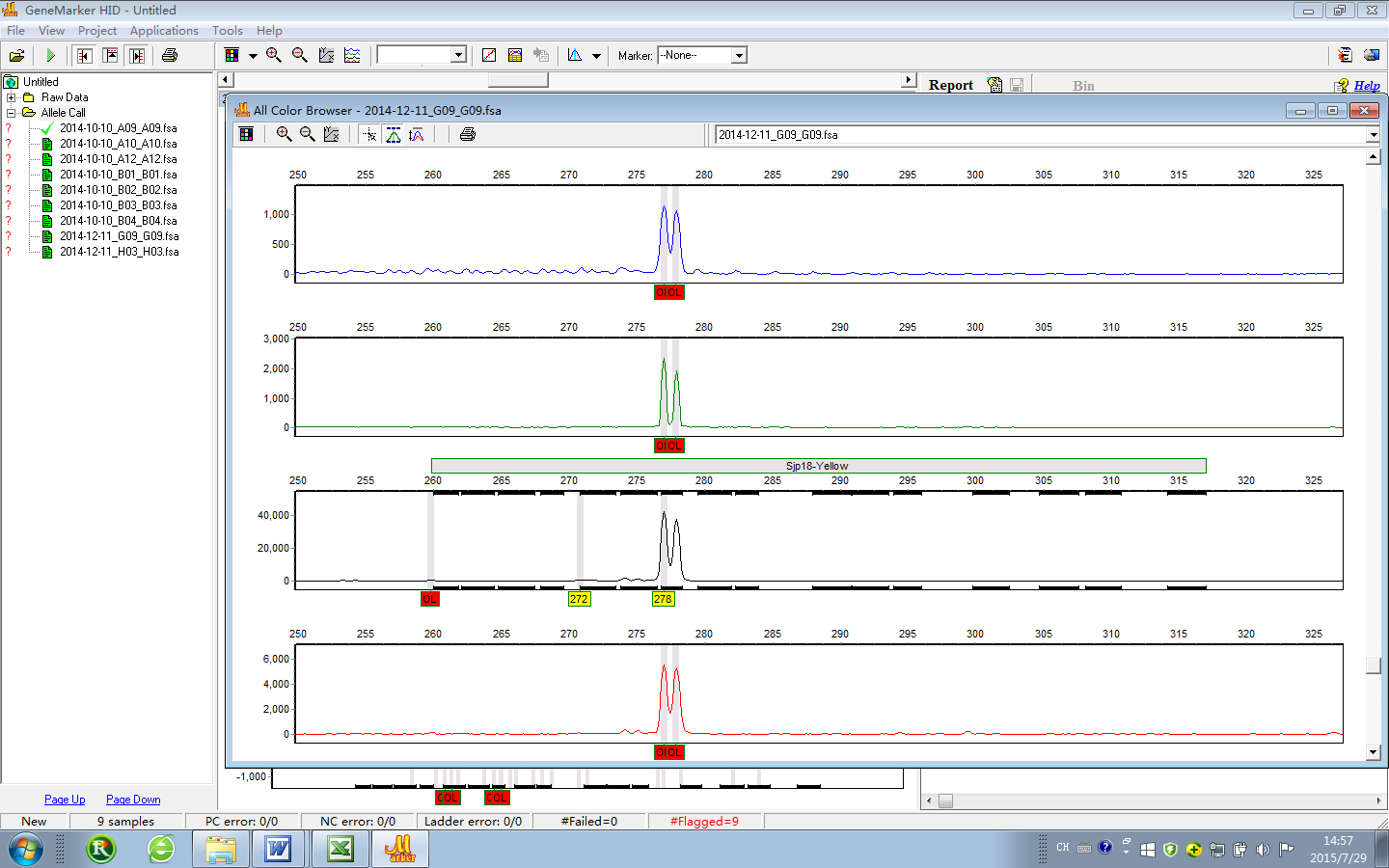


10) C2, female worm: 272, 278


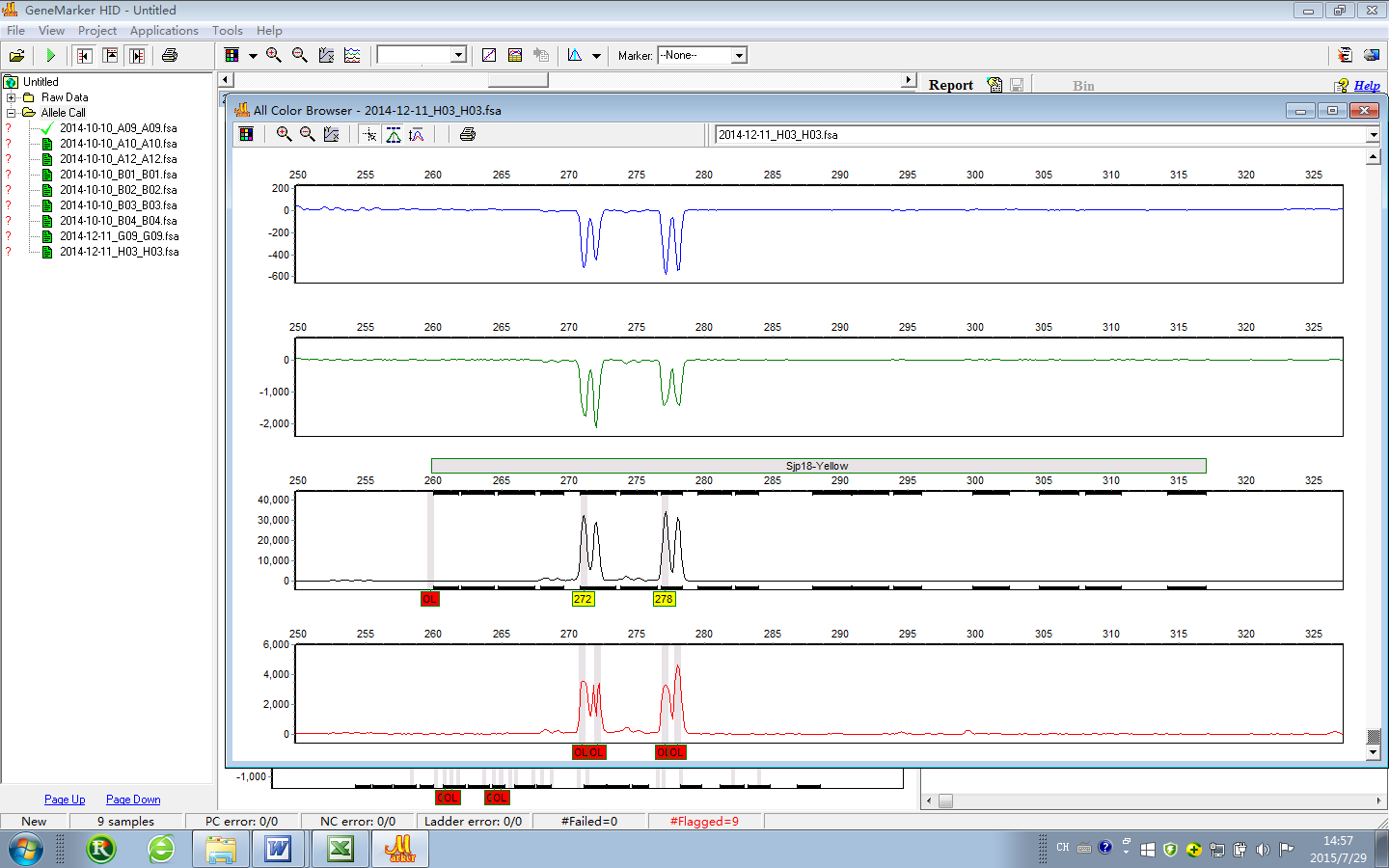


4. Run with Sjp4. Allele calling for B4C2-6, -7, -8, and worms

1) mira B4C2-6: 191, 194


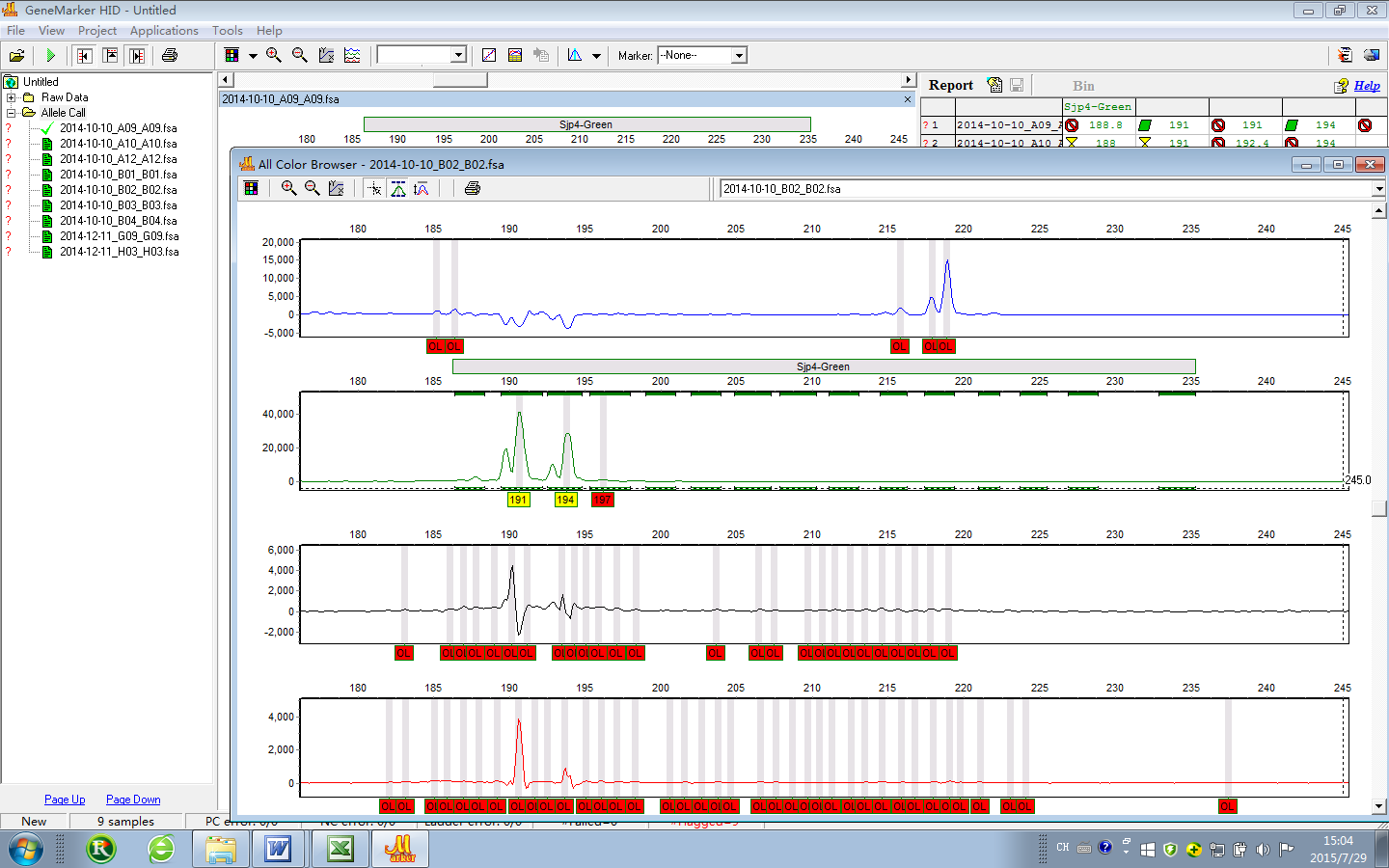


2) mira B4C2-7: 191, 194


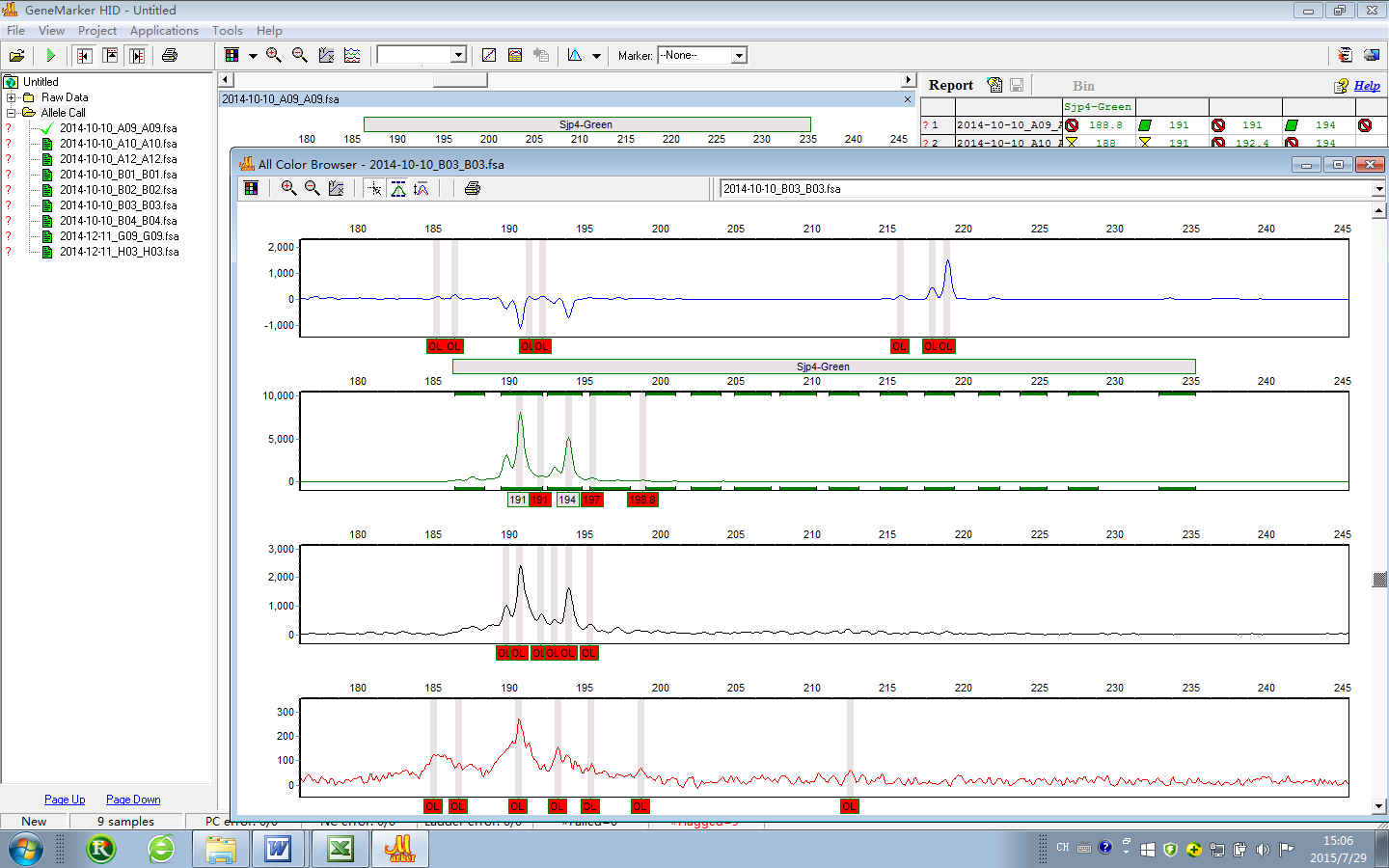
 3) mira B4C2-8: 191, 194


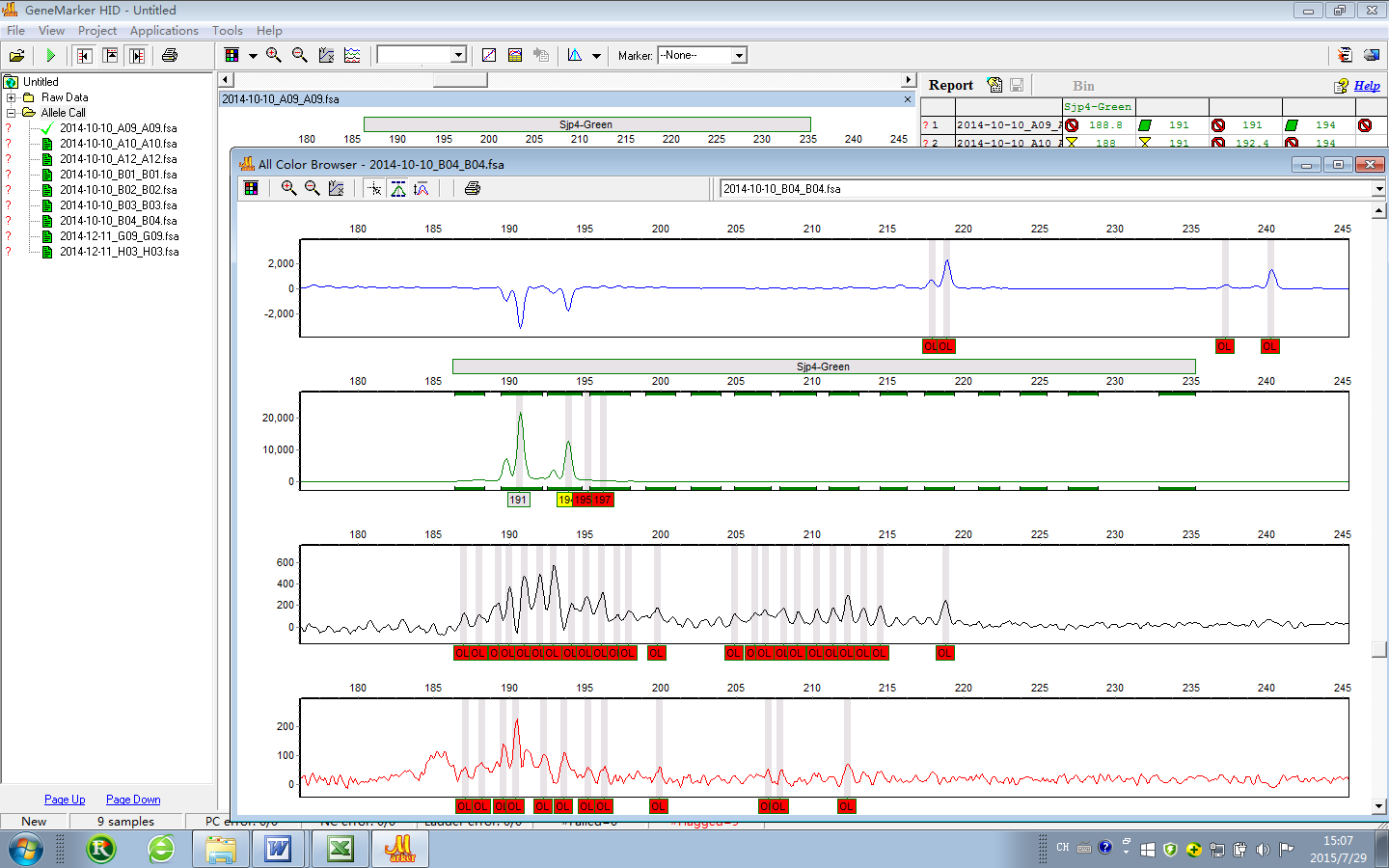


4) B4, female worm: 191, 194


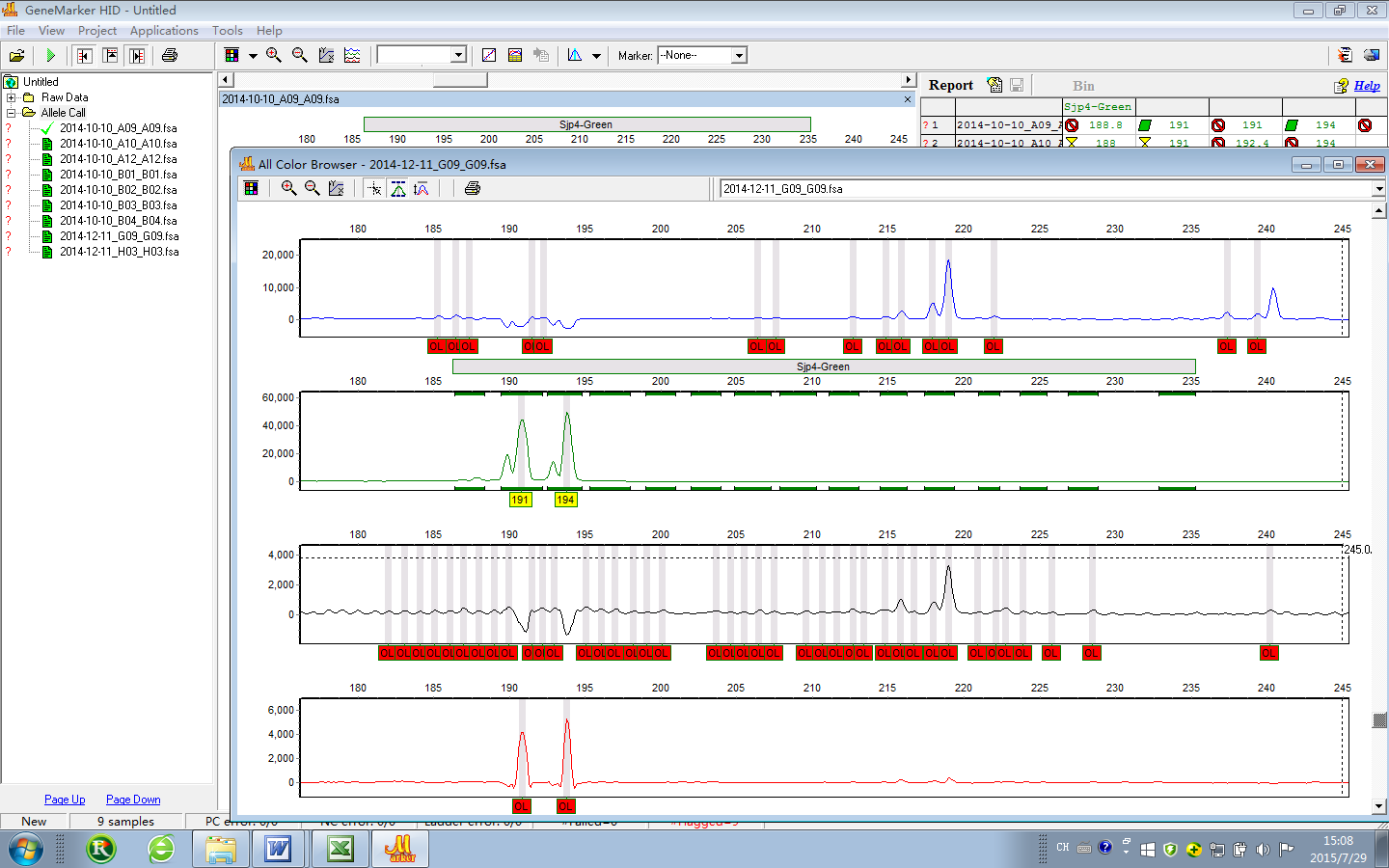


5) C2, male worm: 191, 194


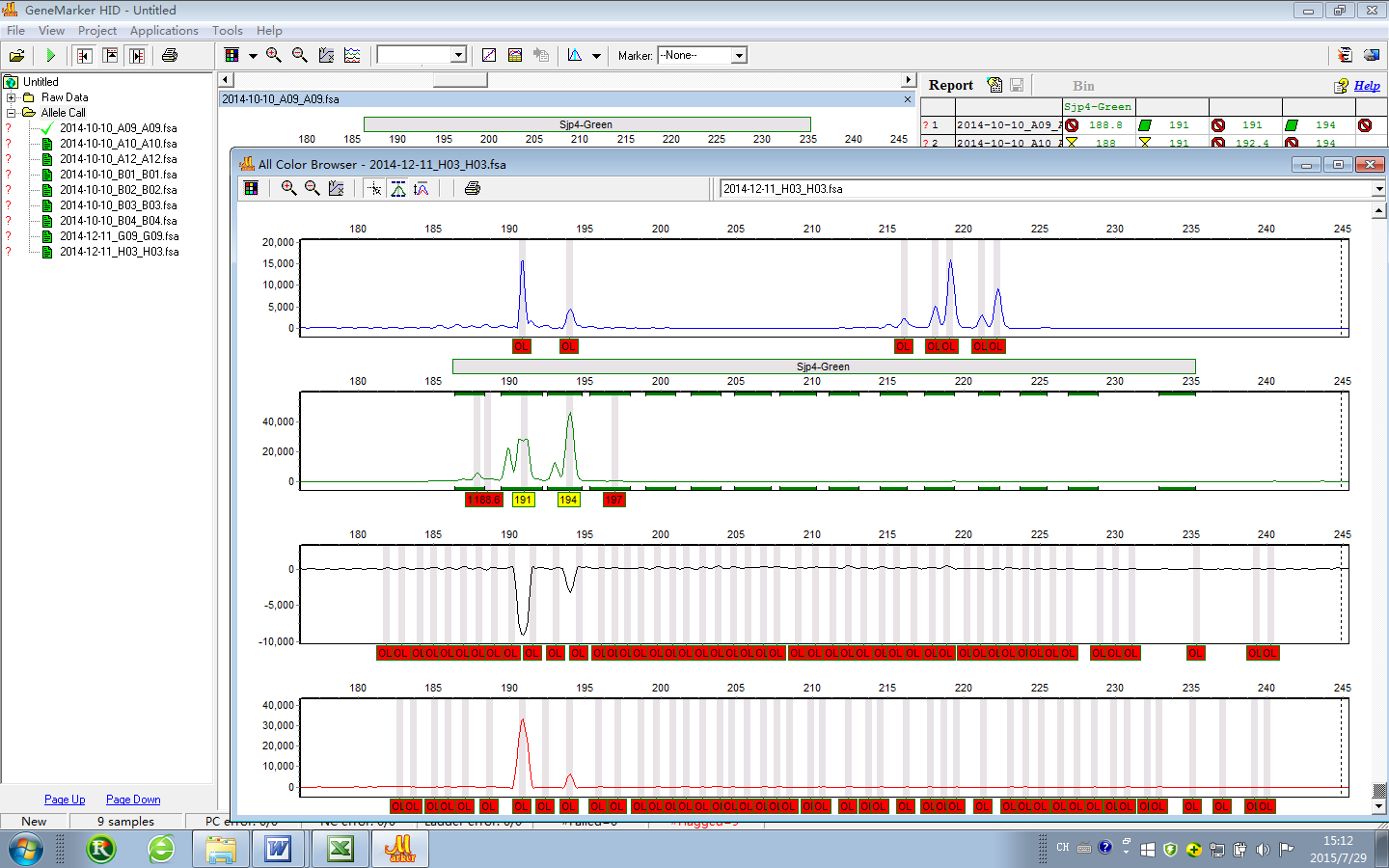


5. Run with Sjp58. Allele calling for B4C2-3, -4, -6, and worms

1) mira B4C2-3: 477, 480


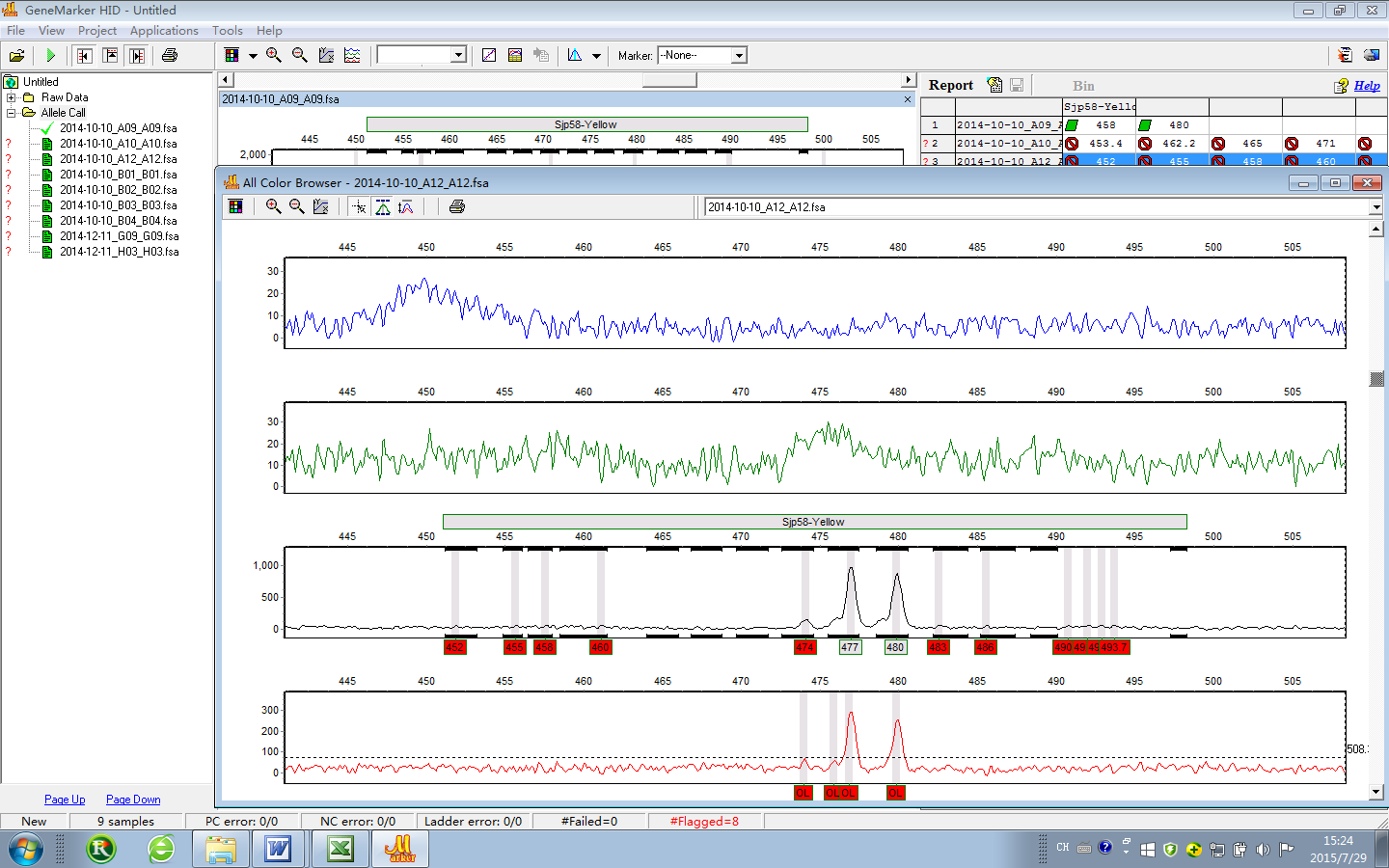


2) mira B4C2-4: 477, 486


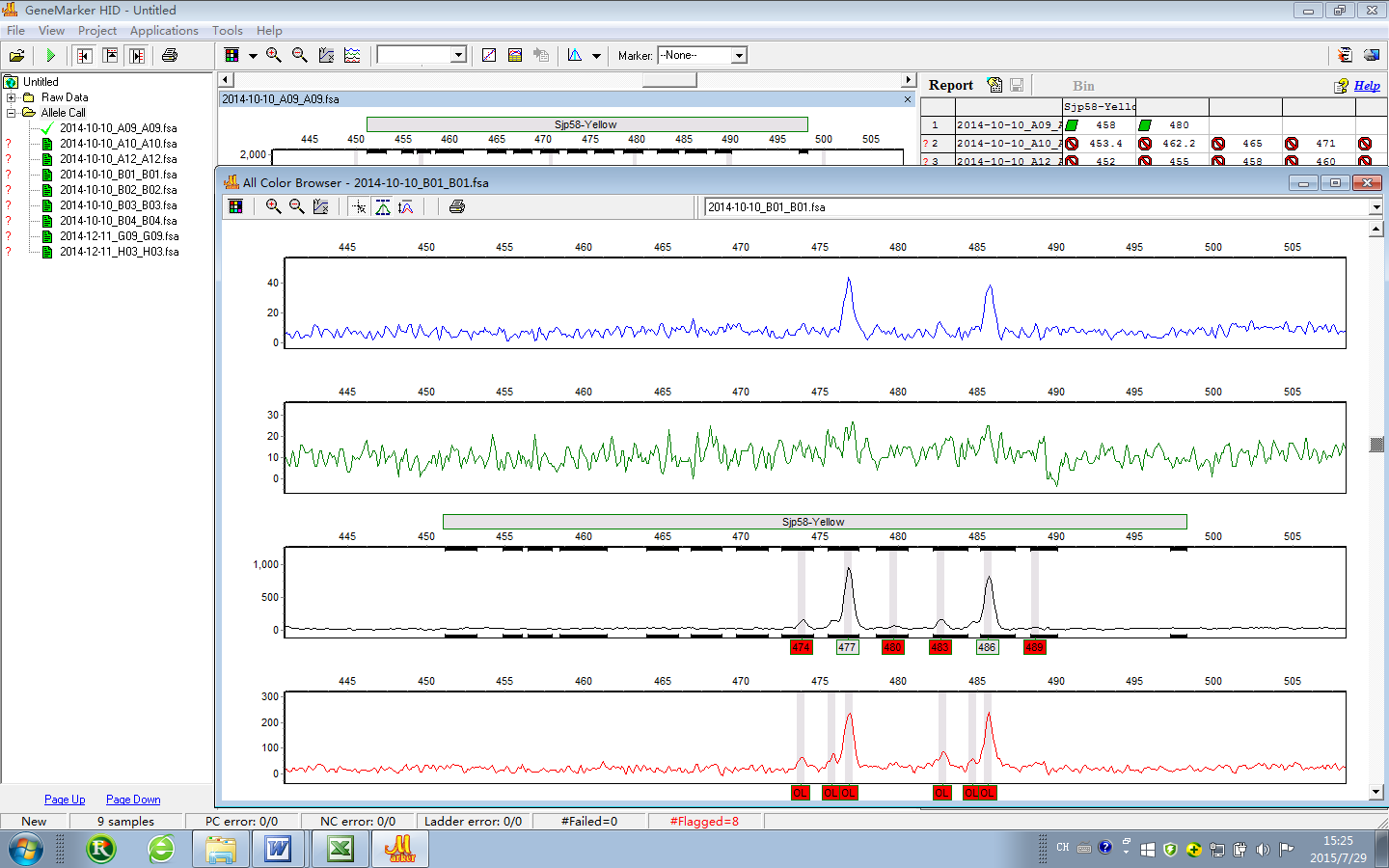


3) mira B4C2-6: 458, 480


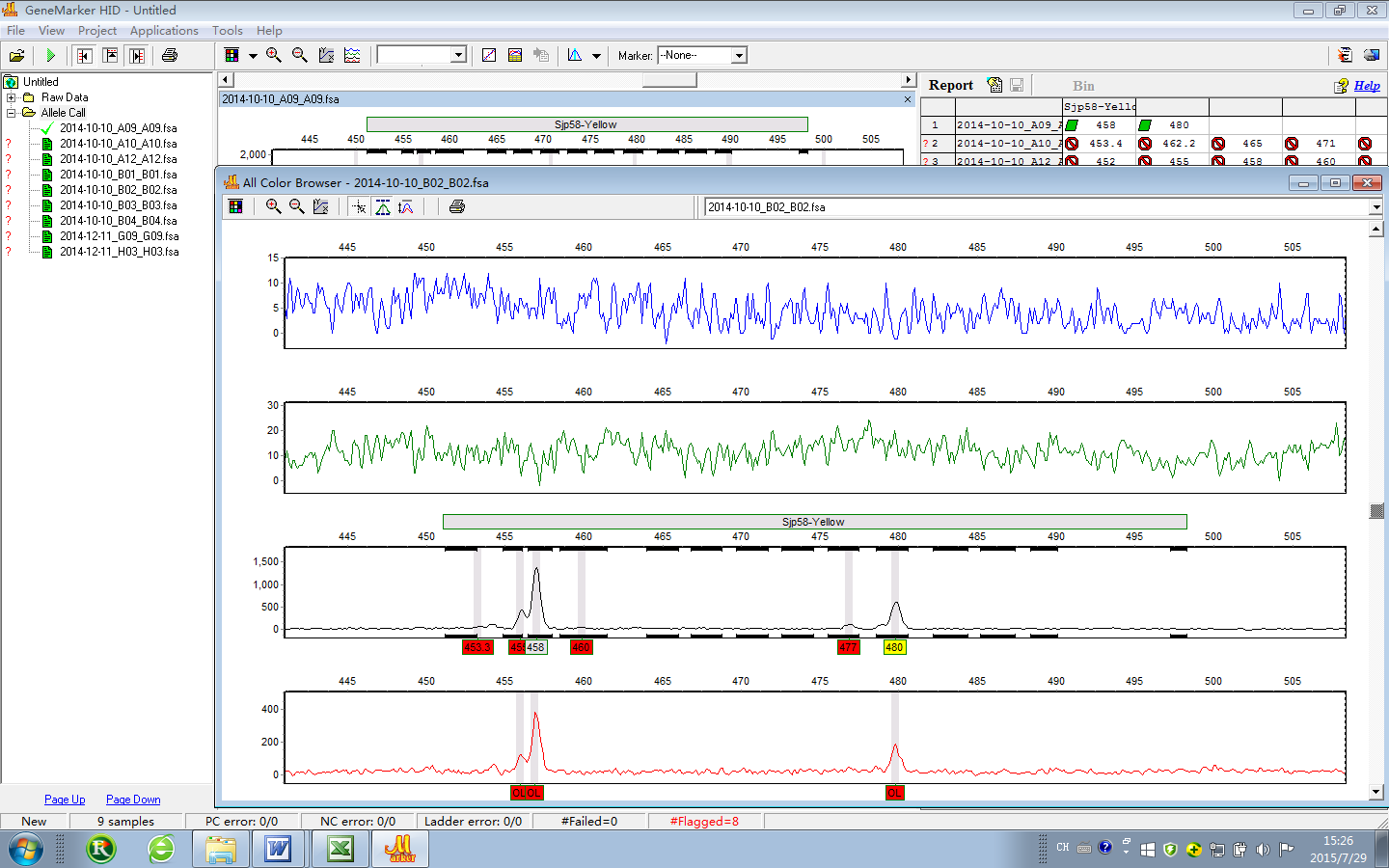


4) B4, female worm: 480, 486


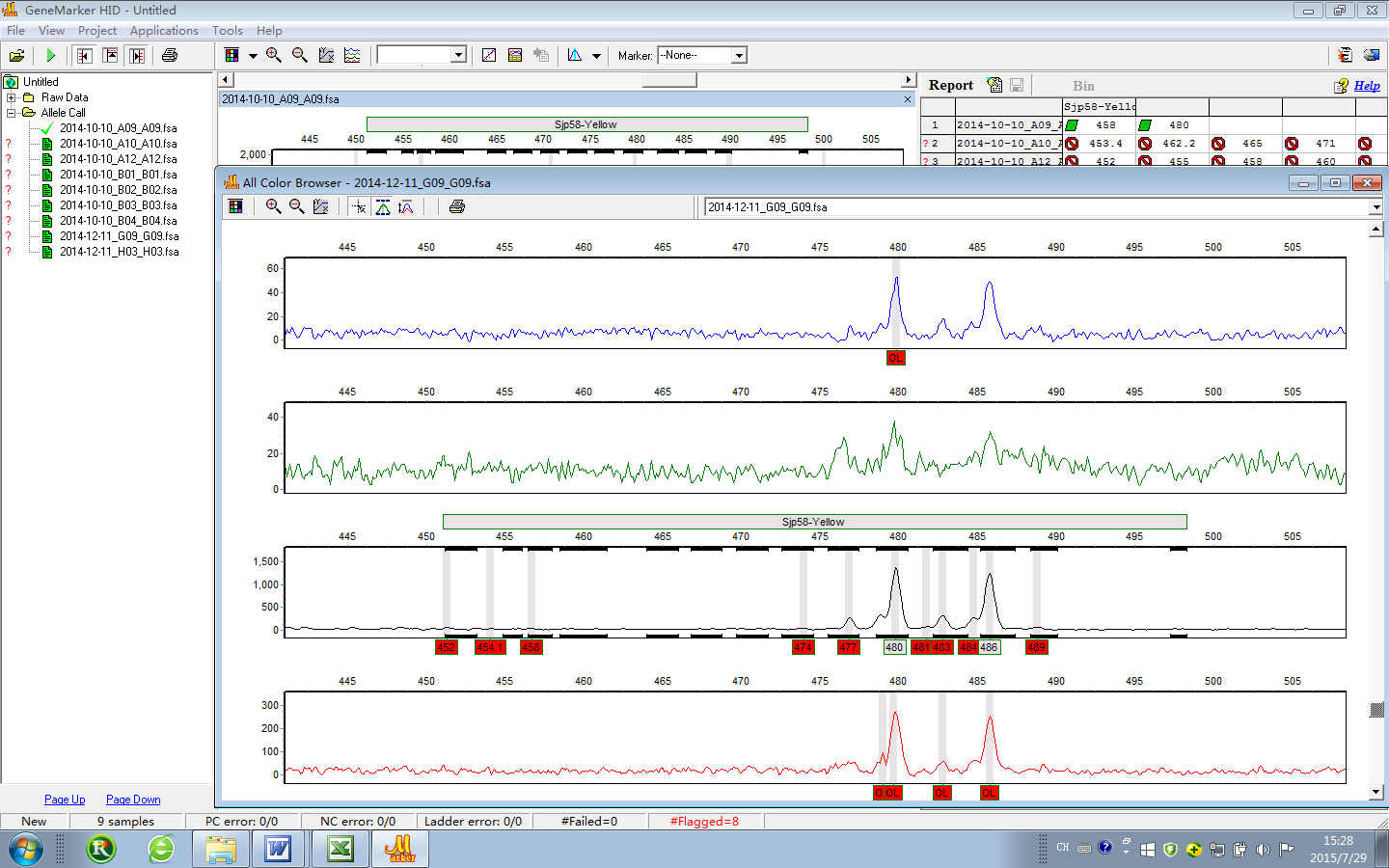


5) C2, male worm: 458, 477


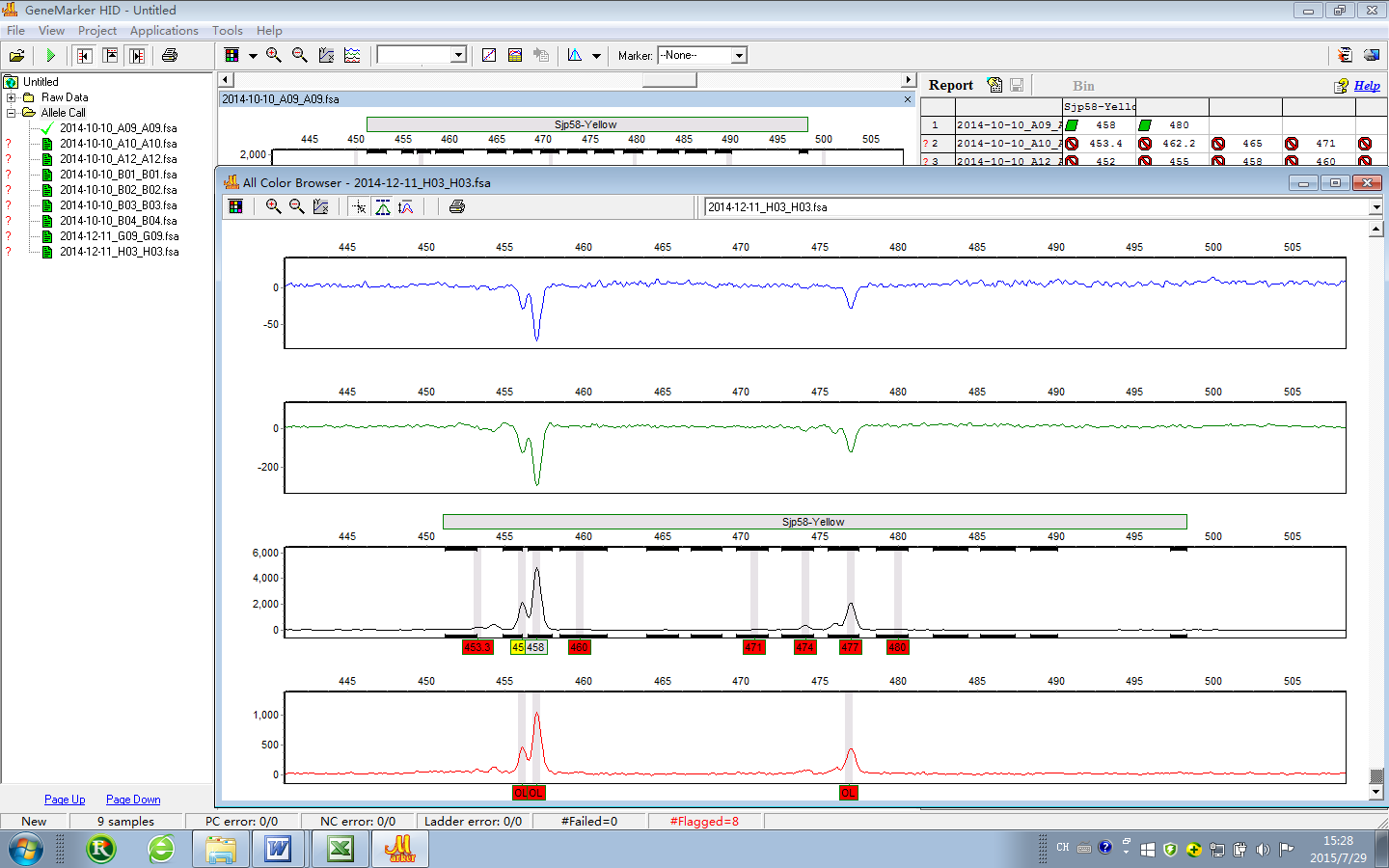


6. Run with Sjp60. Allele calling for B4C2-1, -2, -4, -5, and worms

1) mira B4C2-1: 143, 143 (**incompatible with** its parents B4 + C2)


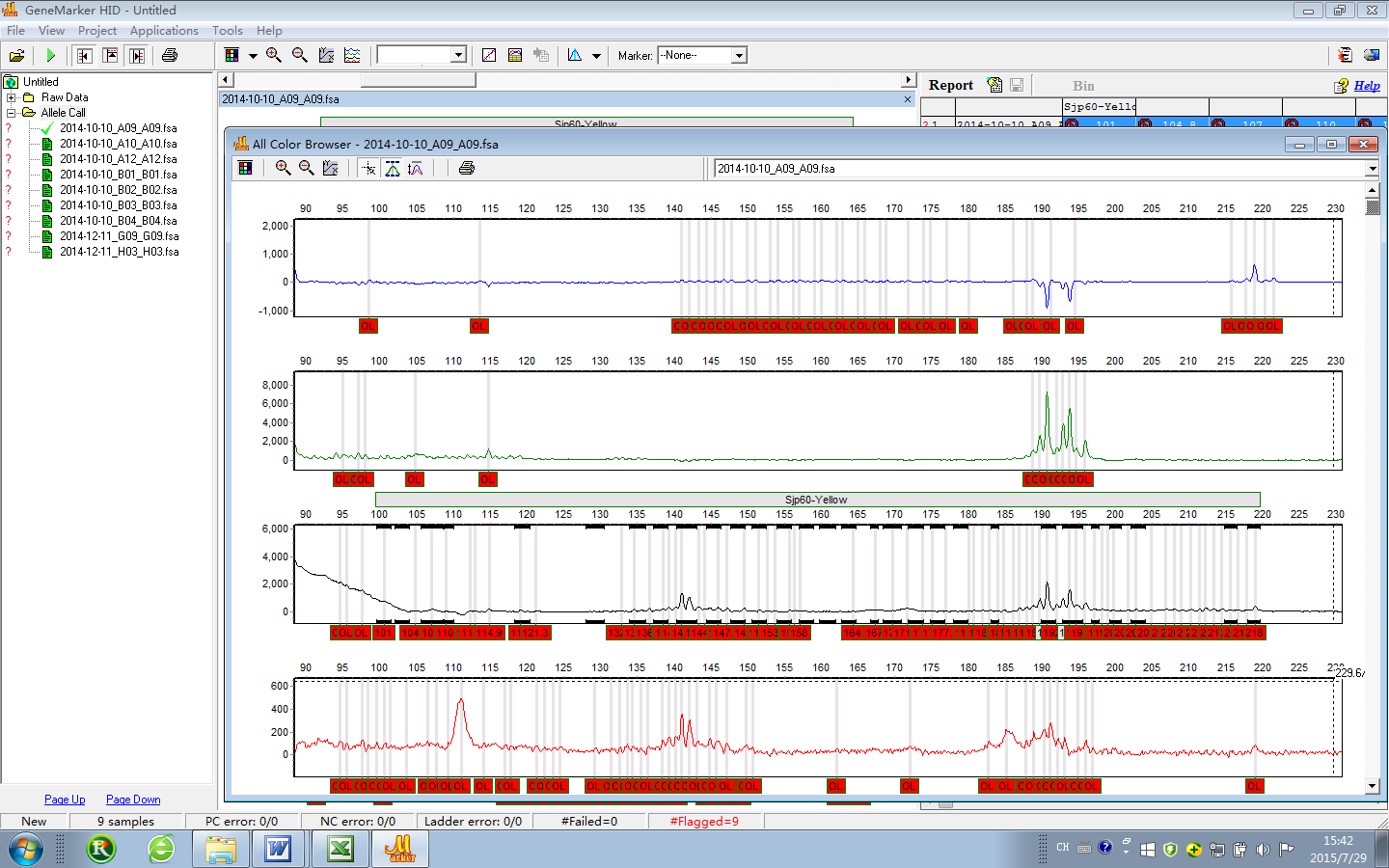


2) mira B4C2-2: 143, 155


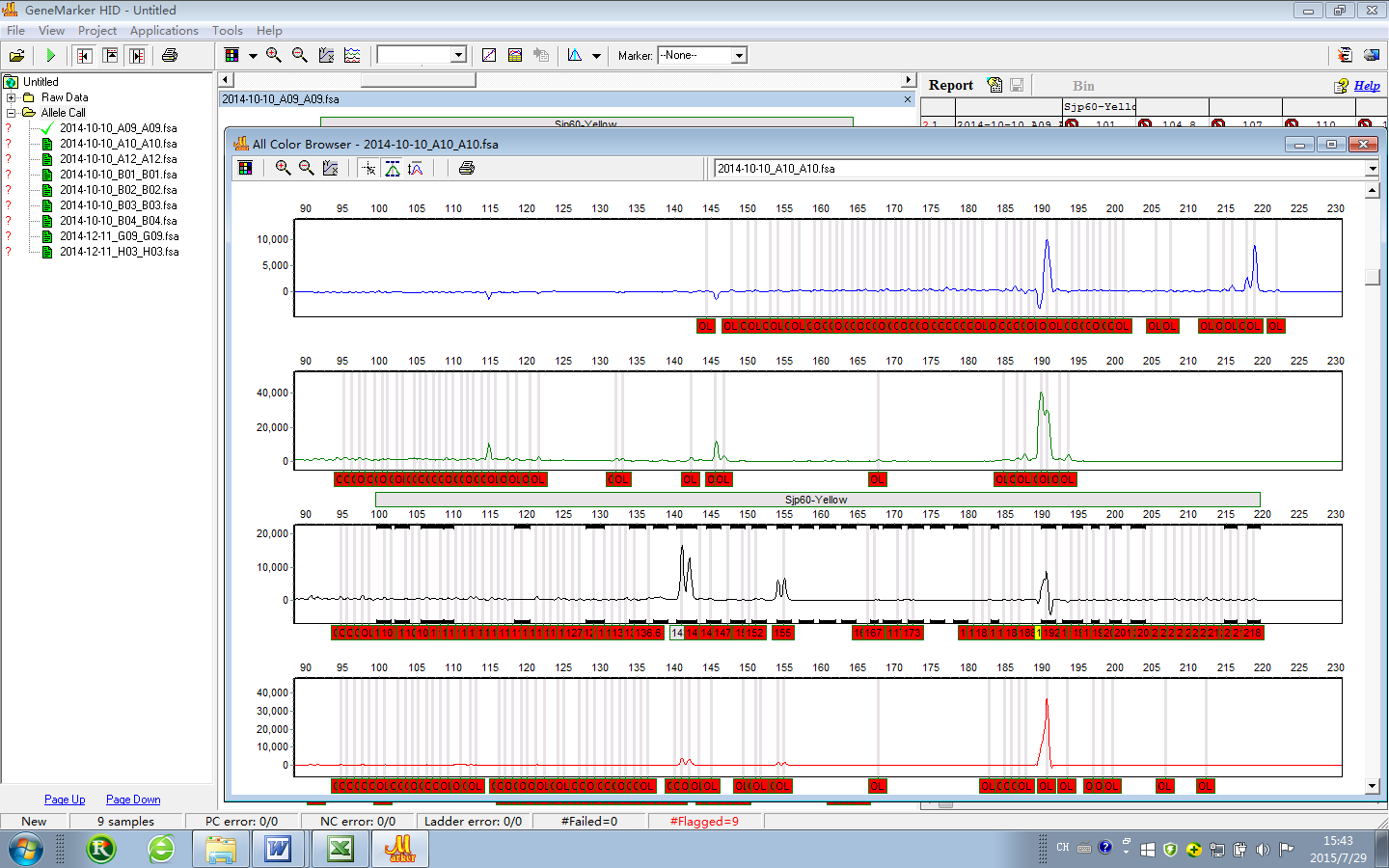


3) mira B4C2-4: 143, 155


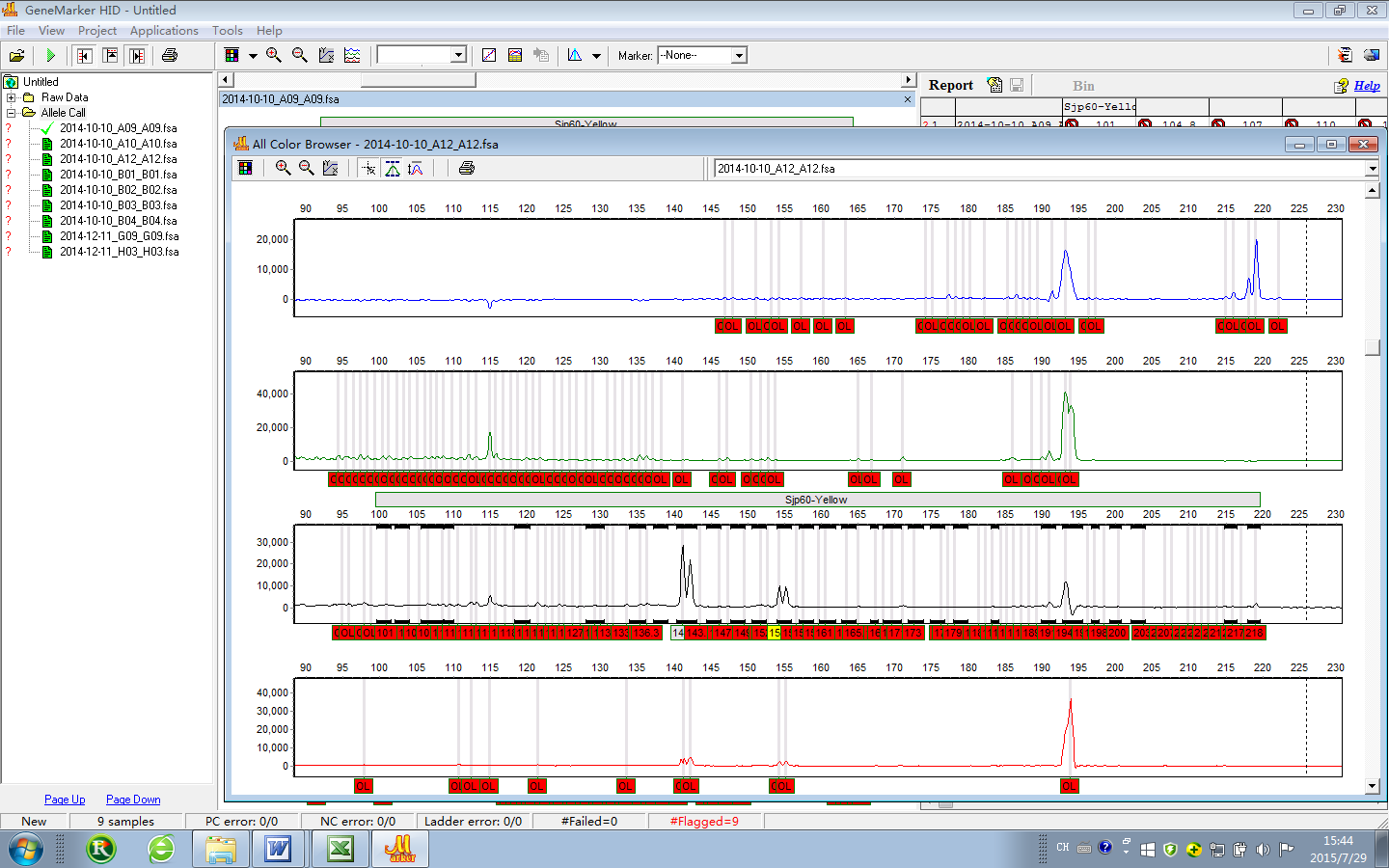


4) mira B4C2-5: 143, 155


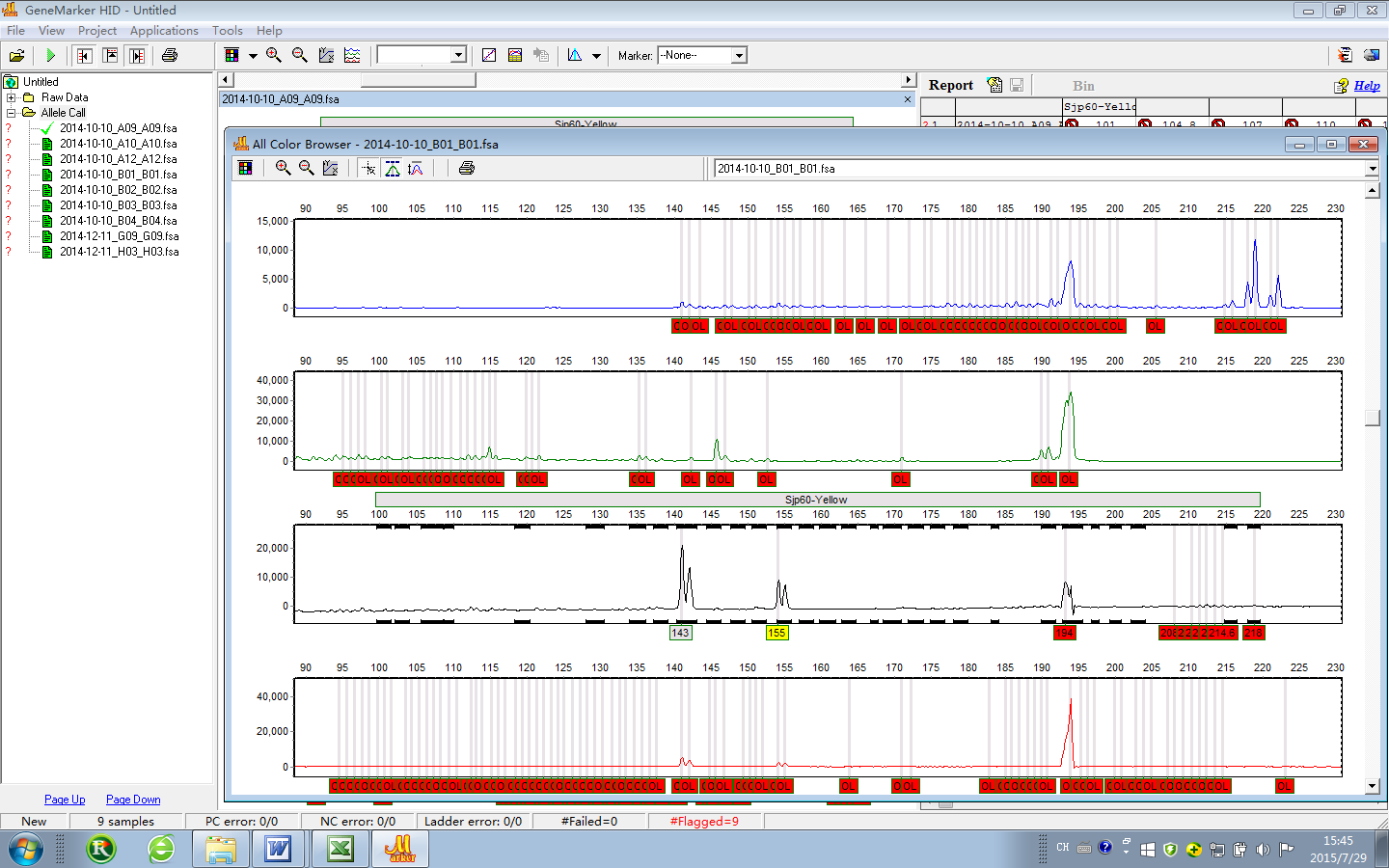


5) mira B4C2-6: 143, 155


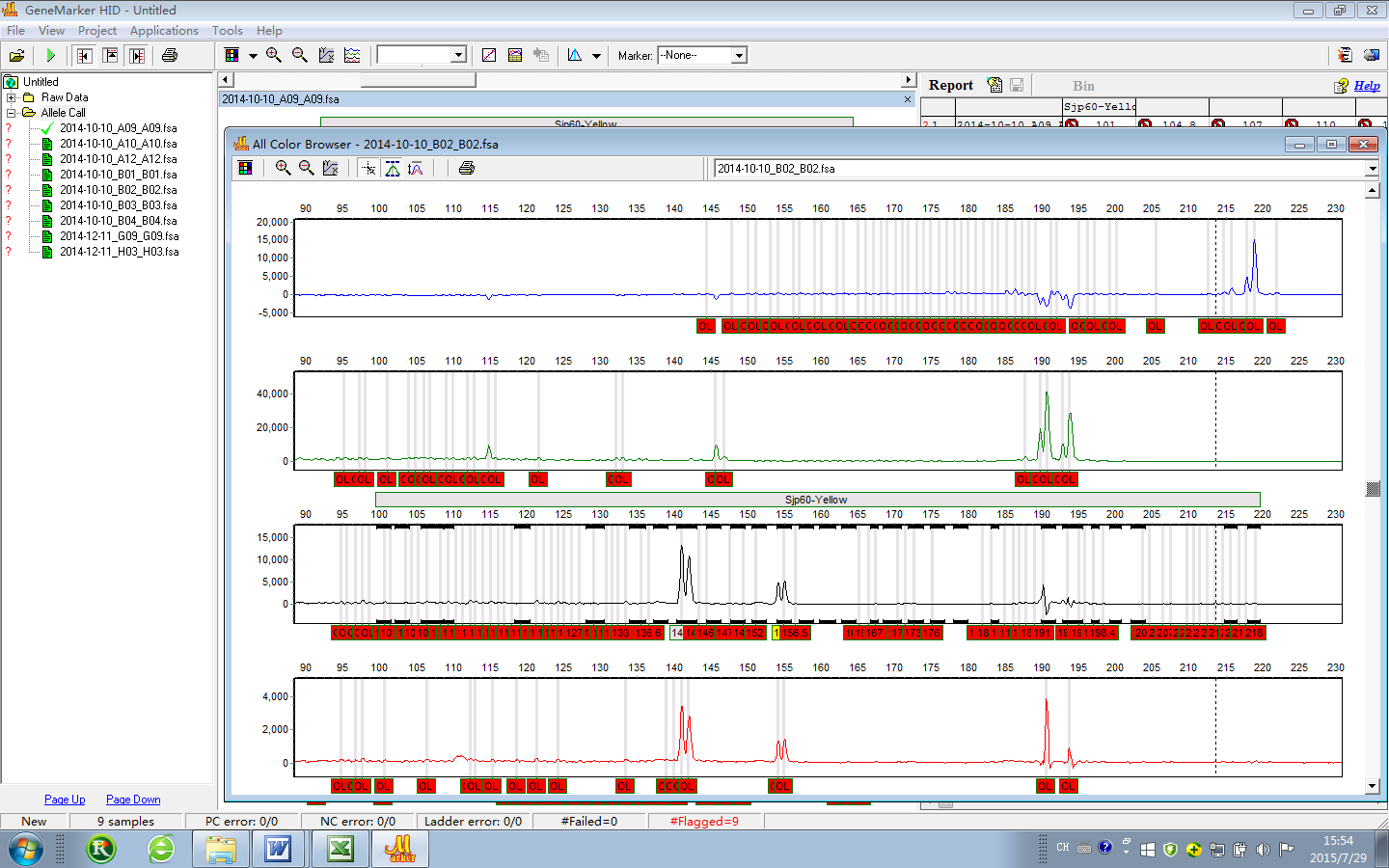


6) mira B4C2-8: 143, 155


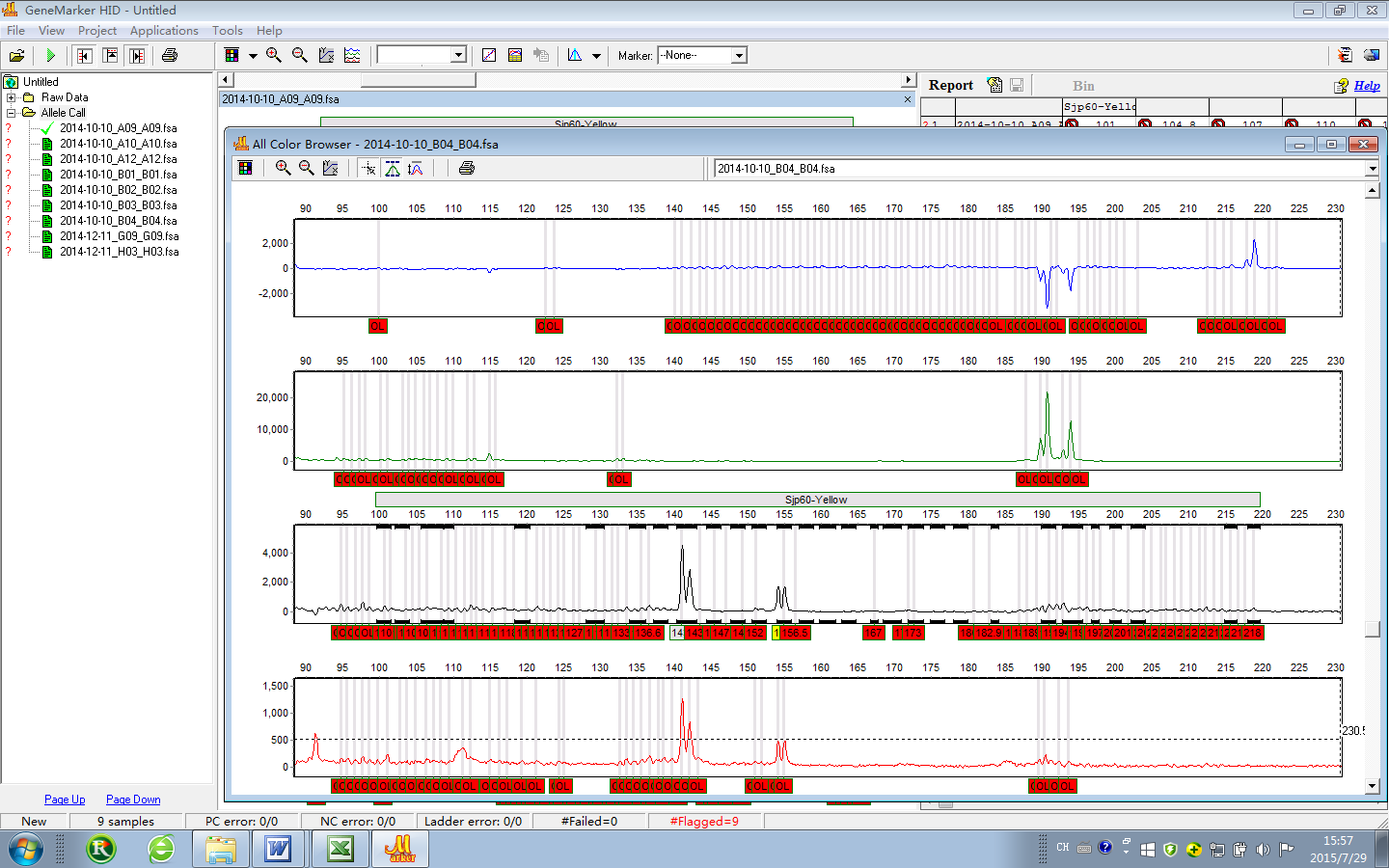
 7) B4, female worm: 155, 155


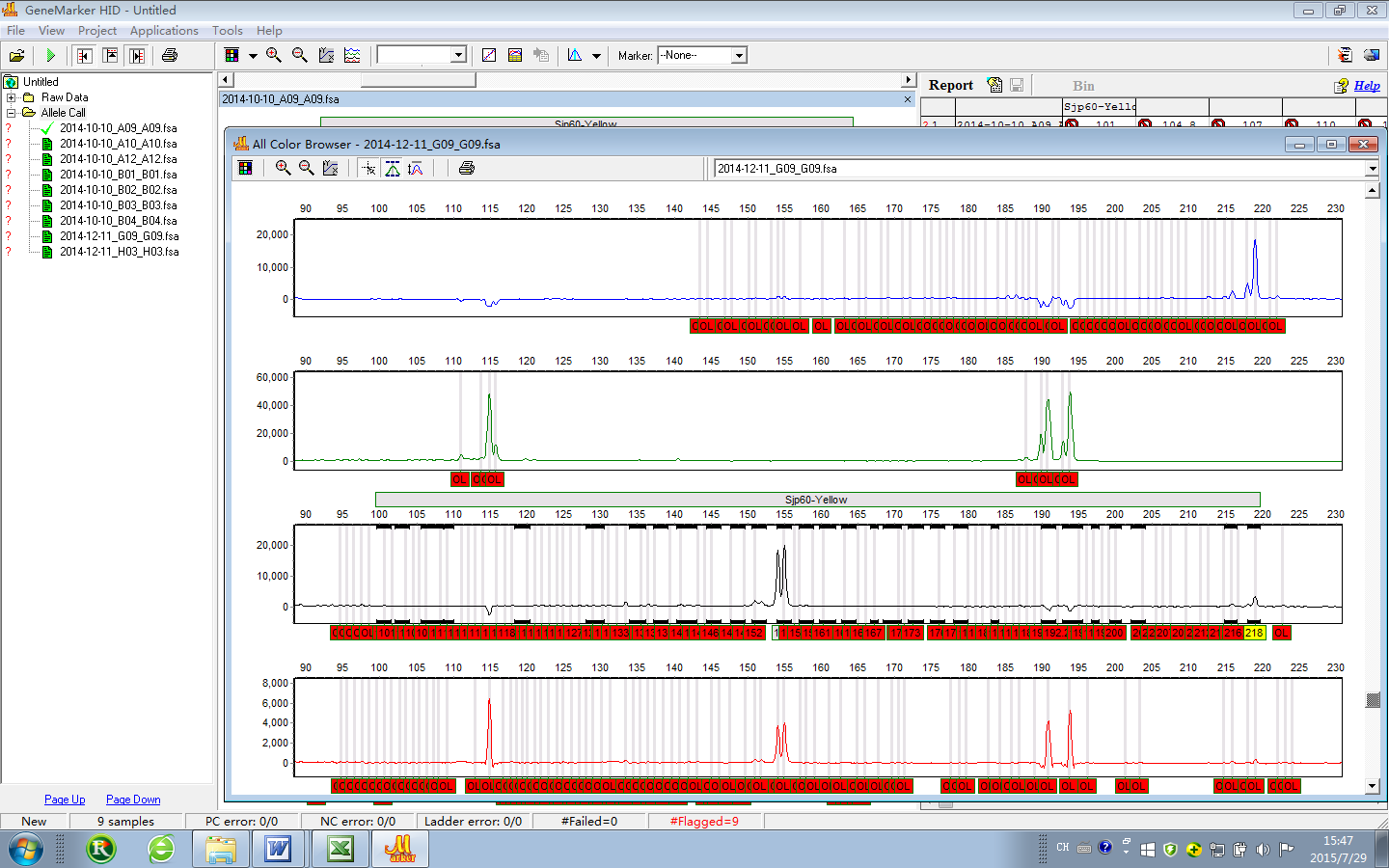


8) C2, male worm: 143, 143


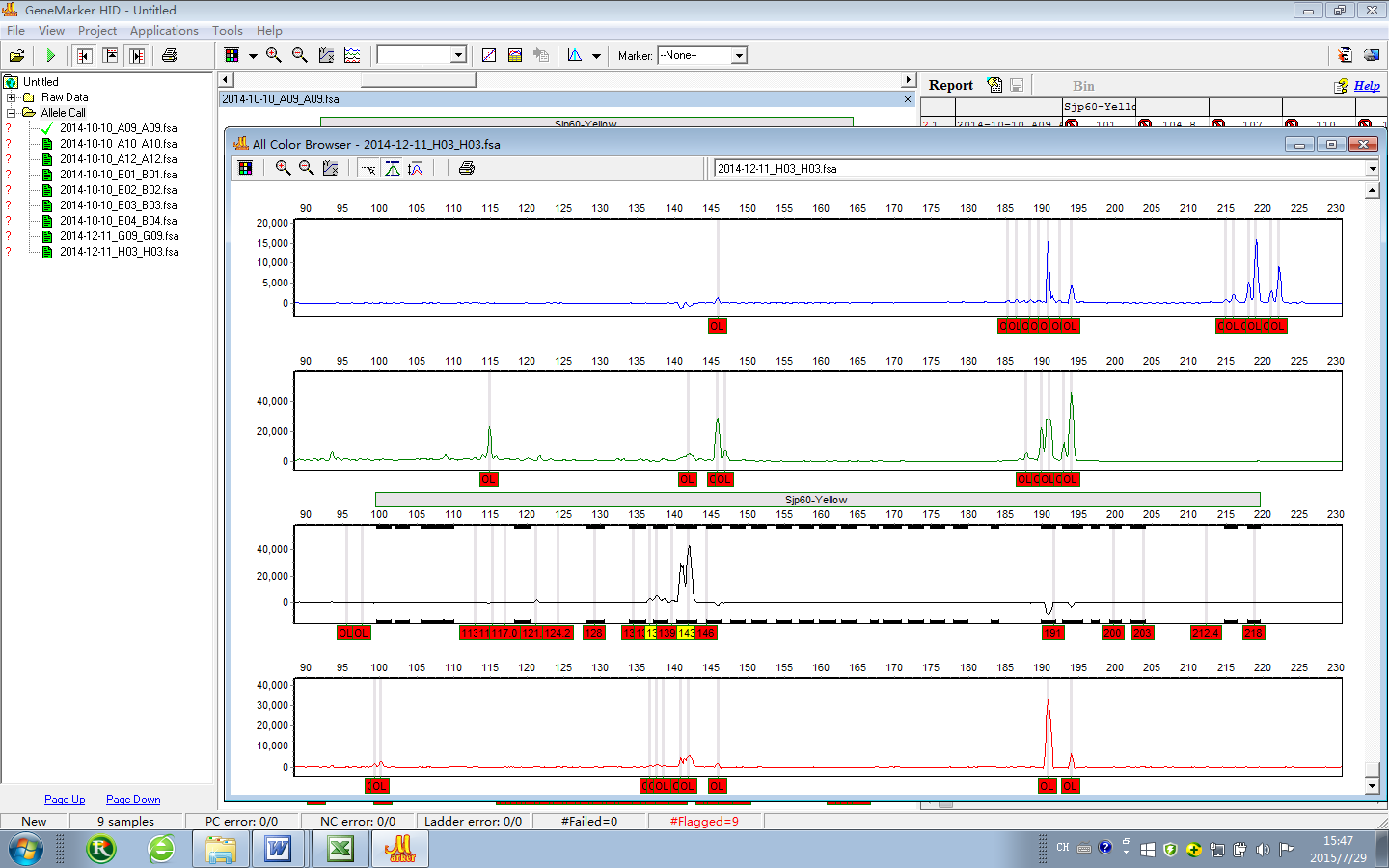

Supplement: Additional file 1: — An example for allele calling with GeneMaker HID V2.6.1 Demo. (DOC 3162 kb) [file 13071_2015_1074_MOESM1_ESM.doc]
